# Linear Atomic Cluster Expansion Force Fields for Organic Molecules: Beyond RMSE

**DOI:** 10.1021/acs.jctc.1c00647

**Published:** 2021-11-04

**Authors:** Dávid Péter Kovács, Cas van der Oord, Jiri Kucera, Alice E. A. Allen, Daniel J. Cole, Christoph Ortner, Gábor Csányi

**Affiliations:** † Engineering Laboratory, University of Cambridge, Cambridge, CB2 1PZUnited Kingdom; ‡ Department of Physics and Materials Science, University of Luxembourg, L-1511Luxembourg City, Luxembourg; ¶ School of Natural and Environmental Sciences, Newcastle University, Newcastle upon Tyne, NE1 7RUUnited Kingdom; § Department of Mathematics, University of British Columbia, Vancouver, BC, CanadaV6T 1Z2

## Abstract

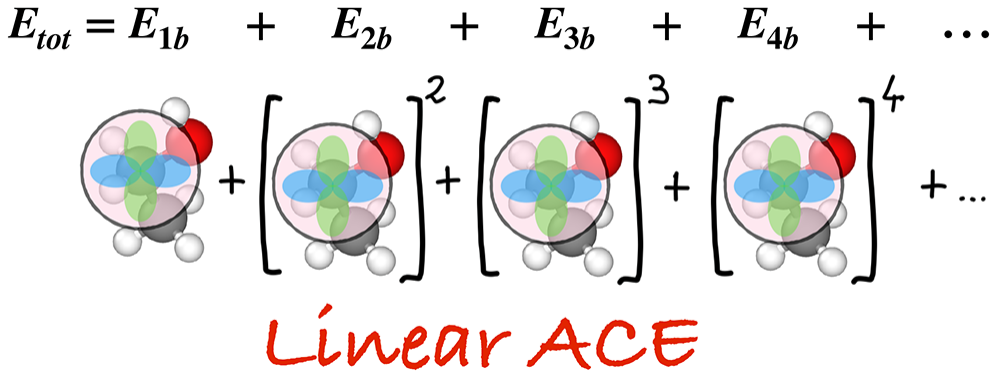

We demonstrate that fast and accurate linear force fields can be built for molecules using the atomic cluster expansion (ACE) framework. The ACE models parametrize the potential energy surface in terms of body-ordered symmetric polynomials making the functional form reminiscent of traditional molecular mechanics force fields. We show that the four- or five-body ACE force fields improve on the accuracy of the empirical force fields by up to a factor of 10, reaching the accuracy typical of recently proposed machine-learning-based approaches. We not only show state of the art accuracy and speed on the widely used MD17 and ISO17 benchmark data sets, but we also go beyond RMSE by comparing a number of ML and empirical force fields to ACE on more important tasks such as normal-mode prediction, high-temperature molecular dynamics, dihedral torsional profile prediction, and even bond breaking. We also demonstrate the smoothness, transferability, and extrapolation capabilities of ACE on a new challenging benchmark data set comprised of a potential energy surface of a flexible druglike molecule.

## 1 Introduction

The efficient simulation of the dynamics of molecules and materials based on first-principles electronic structure theory is a long-standing challenge in computational chemistry and materials science. There is a trade-off between the accuracy of describing the Born–Oppenheimer potential energy surface (PES)^1^ and the length and time scales that are accessible in practice. A convenient way to measure this trade-off is by considering the *total number of simulated atoms*, which can be a result of either generating a few configurations consisting of many atoms or many configurations (e.g., a long molecular dynamics trajectory) each consisting of fewer atoms. Explicit electronic structure simulations are extremely accurate and systematically improvable. They can treat on the order of a million simulated atoms in total using either cubic scaling methods and molecular dynamics or linear scaling algorithms on larger systems. Alternatively, in order to simulate many orders of magnitude more atoms, the PES can be parametrized in terms of the nuclear coordinates only. In this way, the electrons do not have to be treated explicitly, which simplifies the simulations considerably. These methods can routinely model a trillion (10^12^) or more simulated atoms.

When parametrizing the PES, it is natural to decompose the total energy of the system into body-ordered contributions, which can then be resummed into local atomic (or *site*) energies. The site energy of atom *i* is written as
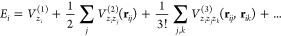
1where indices *j* and *k* run over all neighbors of atom *i* (either unrestricted or within a cutoff distance *r*_cut_), *z*_*i*_ denotes the chemical element of atom *i*, and **r**_*ij*_ = **r**_*j*_ – **r**_*i*_ are the relative atomic positions.

The traditional approach to the parametrization of the body-ordered terms for molecular systems is to use physically motivated simple functional forms with few parameters, leading to “empirical force fields”. These models typically require a predetermined topology, meaning that the parameters describing the interactions of a certain atom depend on its neighbors in the bonding graph that is specified before the simulation and is not allowed to change.^2−5^ The potential energy is then written as a sum of body-ordered bonded and nonbonded terms, for example:
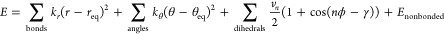
2where *r*, θ, and ϕ describe the intramolecular bond lengths, angles, and dihedral angles in the molecule, and *E*_nonbonded_ contains a Lennard-Jones (LJ) term accounting for van der Waals and short-range repulsive interactions and a Coulomb term to describe the long-range electrostatics. The bonded terms can be made equivalent to the body order in eq 1 by rewriting the sum over atom-tuples into sums over sites. The advantage of the simple functional form of the bonded terms is very fast evaluation and ease of fitting due to the small number of free parameters.^2,6−8^ On the other hand, this simplicity limits the achievable accuracy^9^ and requires significant modification to incorporate reactivity.^10^ Note that while in the most widely used force fields, the nonbonded interactions are two-body, this is not the case for polarizable force fields, such as Amoeba.^11^ Moreover, the direct evaluation of terms beyond three-body contributions is computationally expensive, in general growing exponentially with the body order, which severely limits the possibility of systematically improving force fields by adding higher body order terms.

Over the past 10 years a new approach has emerged, employing machine learning (ML) methods to parametrize the PES. Instead of the body order expansion, the site energy is approximated by a neural network or a Gaussian process regressor (GPR), both of which are extremely flexible functional forms, proven to be universal approximators.^25^ Due to this flexibility there is no need to specify topology or atom types beyond the identity of the chemical element, and much higher model accuracy can be achieved given an appropriate (typically rather large) training set. On the other hand, this flexibility comes also at a cost: there is no guarantee that the behavior of these ML models remains chemically sensible in regions of configuration space where there is not enough training data. Spurious local minima or even wildly wrong atomization energies are par for the course.^26^ The most prominent examples of ML models are atom-centered symmetry function based feed forward neural networks introduced by Behler and Parinello^27^ that also include the family of ANI force fields^17,28^ and DeepMD,^18^ the atomic neighborhood density based GPR models like Gaussian approximation potentials (GAP)^14,29^ and FCHL,^15^ the gradient domain kernel based sGDML,^16^ message passing graph neural network based Schnet,^23^ Physnet,^24^ DTNN,^30^ and DimeNet,^21^ and most recently the covariant or equivariant neural network based Cormorant^22^ and PaiNN.^19^

There is also a third family of methods, which expands the PES as a linear combination of body-ordered symmetric polynomial basis functions. The origins of this approach can be traced back to the work of Bowman and Braams^31,32^ (permutationally invariant polynomials (PIPs)), which approximated the PES of small molecules to extremely high accuracy, albeit with exponential scaling in the number of atoms. Introducing finite distance cutoffs reduces this scaling to linear, and the resulting atomic body-ordered permutationally invariant polynomials (aPIPs) have been shown to achieve high accuracy and better extrapolation compared to the above nonlinear machine-learning-based approaches in both molecular and materials systems.^26,33^ The main limitation of the aPIPs approach is that the evaluation time of the site energy increases quickly with body order, making it essentially impossible to go above body order five (certainly when the five atoms are of the same element). More recently, the atomic cluster expansion (ACE)^34,35^ (and the earlier moment tensor potentials^36^) is a formulation of symmetric polynomial approximations that remove the steep scaling of the evaluation of the site energy with the number of neighbors independently of body order, resulting in highly efficient interatomic potentials for materials.^37^

Table 1 compares the main features of the classical force fields, the machine-learning-based potentials, and the linear atomic cluster expansion force fields. In one sense, the linear ACE constitutes a middle ground between the other two: it retains the chemically natural body order but lifts the limitations of fixed topology and inflexible functional form embodied in eq 2.

**Table 1:** Comparison of Different Force Field Fitting Approaches^a^

	molecular mechanics	machine learning	atomic cluster expansion
functional form	fixed	flexible	polynomial
parametrization	nonlinear	nonlinear	linear
no. of parameters	100s	10^4^–10^5^	10^4^–10^5^
body-ordered?	yes	no	yes
topology-free?	no	yes	yes
fit to	QM and experiment	QM only	QM only

a Molecular mechanics (e.g., AMBER,^2^ CHARMM,^12^ and OPLS^13^), machine learning: kernels (GAP,^14^ FCHL,^15^ and sGDML^16^), and neural networks (ANI,^17^ DeepMD,^18^ PaiNN,^19^ GMsNN,^20^ DimeNet,^21^ Cormorant,^22^ Schnet,^23^ and Physnet^24^).

The purpose of the present paper is to demonstrate the performance of linear ACE force fields for small organic molecules. After briefly reviewing the general ACE framework and outlining the necessary choices that go into fitting our linear models, we start with the MD17^38^ and ISO17^23^ benchmark data sets. We are particularly interested in going *beyond the RMSE* (or MAE) of energies and forces (the typical target of the loss function in the fit) because practically useful force fields have other desirable properties too: chemically sensible extrapolation, good description of vibrational modes, and accuracy on trajectories self-generated with the force field, just to name a few. The insufficient nature of mean error metrics has been pointed out before.^39−41^ In addition to the above data sets, we also demonstrate the use of ACE on a slightly larger, significantly more flexible molecule that is more representative of the needs of medicinal chemistry applications.

The program of tests as we outlined is designed to explore the capabilities and properties of different approaches to making force fields. We emphasize here that we are not making or testing force fields that are in and of themselves generally useful to others. That is a significant undertaking, and it is to be attempted once we better understand these capabilities and properties and are able to select which approach has the best prospects. Therefore, in addition to quoting literature results for recently published ML schemes, we refit a number of them, where the necessary software is available (sGDML, ANI, and GAP in particular), so that we can show their performance on our tests. We also refit a classical empirical force field (eq 2) to exactly the same training data to more rigorously quantify the anticipated accuracy gains of the ML and ACE approaches.

## 2 Methods

### 2.1 Atomic Cluster Expansion Basis Functions

The atomic cluster expansion (ACE) model^34,35^ keeps the body ordering of terms defined in eq 1 but reduces the evaluation cost by eliminating the explicit summation over atom-tuples. This is accomplished by projecting the atomic neighbor density onto isometry-invariant basis functions. This idea, detailed below, is referred to as the “density trick” and was introduced originally to construct the power spectrum (also known as SOAP) and bispectrum descriptors^14,42^ (which are in fact equivalent to the three- and four-body terms in ACE, respectively, so in a sense the ACE invariants can be considered a generalization of these to arbitrary body order).

**Figure 1:**
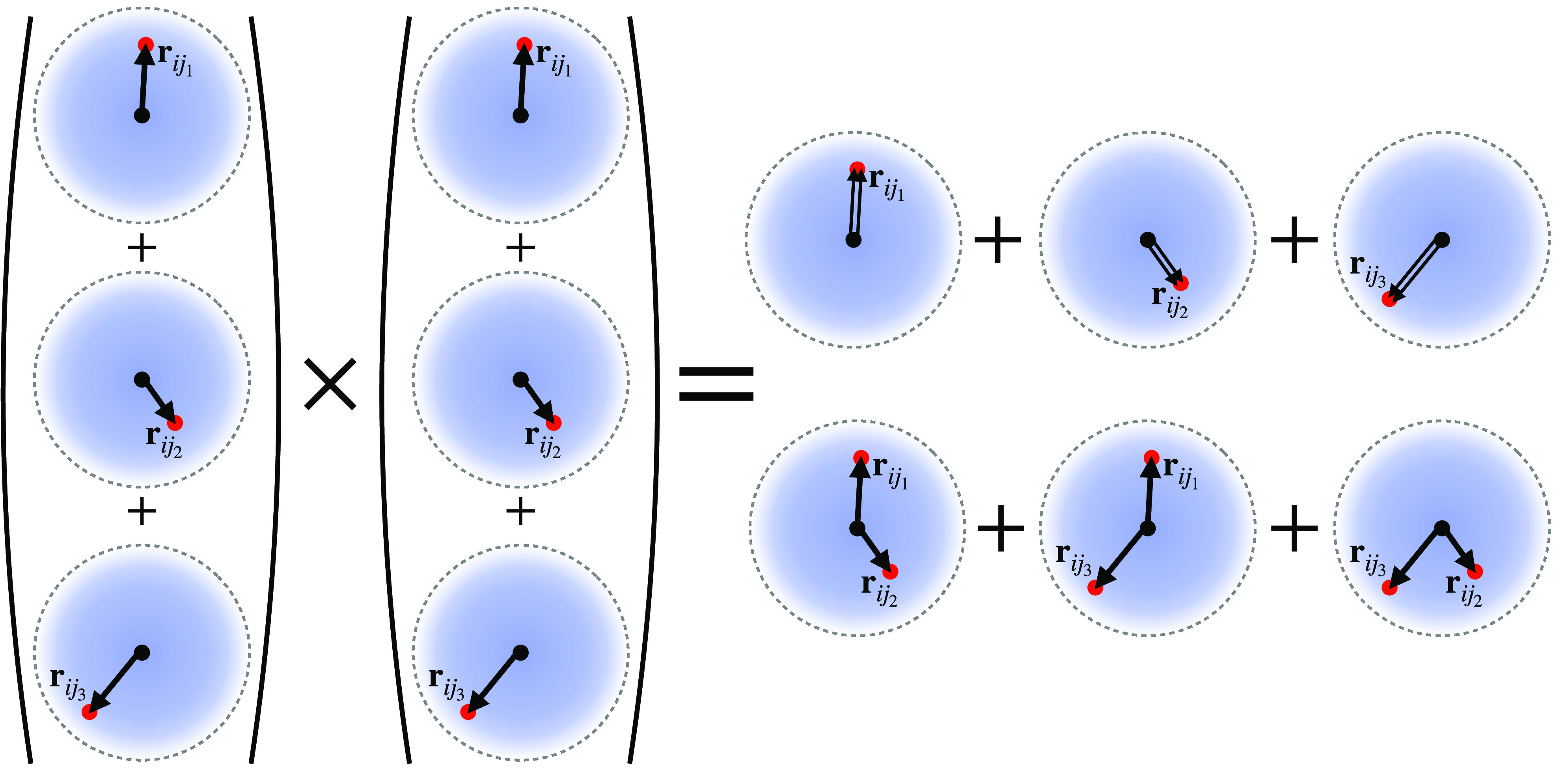
Construction of high-body-order invariant basis functions. A graphical illustration showing how higher body order basis functions can be constructed as products of the projected neighborhood density. The evaluation cost of the basis functions scales linearly with the number of neighbors rather than exponentially by doing the density projection first and then taking the products to obtain higher order basis functions. The figure (and expression) also makes explicit the occurrence of self-interaction terms in the ACE basis. They are automatically corrected through the inclusion of lower order correlations in the basis.

We start by defining the neighborhood density of atom *i* as
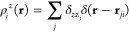
3where ρ_*i*_^*z*^ denotes the density of atoms of element *z* in the neighborhood of atom *i*. This density is projected onto a set of *one-particle basis functions*, which we choose to be a product of a radial basis and real spherical harmonics:
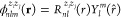
4Here the “one-particle” refers to the single sum over neighbors, with the central atom *i* serving as the center of the expansion. There is considerable flexibility in the choice of the radial basis; the specifics for this work are documented at the end of this subsection. We then define the *atomic base* as the projection of the neighborhood density onto the one-particle basis functions
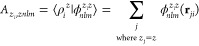
5where the index *z*_*i*_ refers to the chemical element of atom *i*. For notational convenience, we collect the rest of the one-particle basis indices into a multi-index
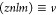
6From the atomic base *A*_*z*_*i*_*v*_, we obtain permutation-invariant basis functions, which we will call the “*A*-basis”, by forming the products
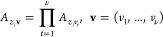
7The product containing ν factors gives a basis function that is the sum of terms each of which depends on the coordinates of at most ν neighbors, and we refer to it either as a ν-correlation or as a (ν + 1)-body basis function (the extra +1 comes from the central atom *i*). A graphical illustration of this construction is shown in Figure 1 for the special case where the two factors are the same. For many (different) factors, taking products of the atomic base (left side of Figure 1) takes a lot less time to evaluate than the explicit sum of all possible products (right side of Figure 1). This is the key step that we referred to as the density trick.

The *A*-basis is not rotationally invariant. We therefore construct a fully permutation and isometry-invariant overcomplete set of functions, which we call the *B*-basis (technically not a basis but a spanning set), by averaging the *A*-basis over the three-dimensional rotation group, O(3)
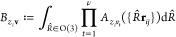
8
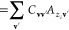
9where the matrix of Clebsch–Gordan coupling coefficients *C*_**vv**′_ is extremely sparse. Many of the resulting basis functions will be linearly dependent (or even zero), but it is relatively straightforward to remove these dependencies in a preprocessing step to arrive at an actual basis set. We refer to Dusson et al.^35^ for the details of the procedure outlined up to this point.

The *B*-basis in eq 8 is complete in the sense that any function of the neighboring atoms that is invariant to permutations and rotations can be expanded as a linear combination of the basis functions. We therefore write the site energy of ACE as
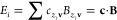
10The above equation makes it clear that the model is linear in its free parameters, the *c*-coefficients. The *B*-basis functions are polynomials of the atomic coordinates, and in order to show that the explicit body ordering has been retained, we can switch back to using the *A*-basis (with the product explicitly written out)
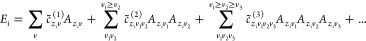
11where the *c̃* can be obtained as linear combinations of the *c*-coefficients appearing in eq 10, using the transformation defined in eq 9.

Now the body ordering is readily identified. Each term corresponds precisely to a sum of ν-correlations, i.e., (ν + 1)-body terms as in the traditional body order expansion, eq 1. In practice, we use a recursive scheme^35^ that leads to an evaluation cost that is O(1) per basis function, independent of body order. The number of basis functions does grow with body order, at a rate that has an exponent ν.

The construction outlined so far yields infinitely many polynomials *B*_*z*_*i*_**v**_, which can be characterized by their correlation order ν and their (modified) polynomial degree *D* = ∑_*t*_^ν^*n*_*t*_ + *w*_*Y*_*l*_*t*_, where *n*_*t*_ and *l*_*t*_ come from the multi-index *v*_*t*_ and the weight *w*_*Y*_ is used to trade-off the radial and angular resolution of the basis set. When it comes to defining a model, in practice the expansion is truncated both in the body order and in the maximum polynomial degree at each body order.

### 2.2 Choice of Radial Basis

In the models in this paper we will not use much of the flexibility of the ACE framework, and we simply take *R*_*nl*_^*z*_*i*_*z*_*j*_^(*r*) = *R*_*n*_(*r*), where
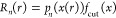
12*r* → *x*(*r*) is a one-dimensional radial transformation, *f*_cut_ is a cutoff or envelope function, and *p*_*n*_ are orthogonal polynomials. For the radial transform we take
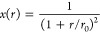
13which amplifies the effect of neighbors closer to the central atom. For the cutoff function we specify both inner and outer cutoffs, *r*_in_ < *r*_out_, and define
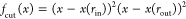
14The polynomials *p*_*n*_ are then defined recursively by specifying that *p*_0_(*x*) = 1, *p*_1_(*x*) = *x*, and the orthogonality requirement
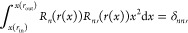
15where we have used the *inverse* of the radial transform, *x* → *r*(*x*). Equation 15 implies that the radial basis *R*_*n*_ and not the polynomials *p*_*n*_ is orthonormal in *x*-coordinates.

The introduction of an inner cutoff is necessary to prevent wildly oscillating behavior in high-energy regions of configuration space where pairs of atoms are very close to one another and little or no training data is available. Alternatively, one could introduce such training data, but that would unnecessarily complicate the construction of training data sets, and this inner cutoff mechanism is sufficient. To ensure short-range repulsion, we augment the large multibody ACE basis by a small auxiliary basis set, consisting only of low-polynomial-degree pair interaction (two-body) functions. The construction is exactly the same as before, but we change the cutoff function to
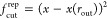
16

### 2.3 Basis Selection

Before we can parametrize the ACE force field, we need to select a specific finite basis set chosen from the complete ACE basis constructed in the previous section. There are three approximation parameters: the cutoff radius (*r*_cut_ = *r*_out_), the maximum correlation order ν^max^, and the maximum polynomial degrees *D*_ν_^max^ corresponding to order ν basis functions. We have already specified the cutoff radius in the definition of the radial basis in eq 12. The basis is then chosen as (a linearly independent subset of) all possible basis functions *B*_*i***v**_ with correlation order at most ν^max^ and polynomial degree at most *D*_ν_^max^.

In all models for molecules with three or fewer distinct elements, we take ν^max^ = 4, which corresponds to a general five-body potential. In models for molecules with four or more distinct elements, we reduce this to ν^max^ = 3 (four-body potential). The weight *w*_*Y*_ specifies the relative importance of the radial and angular basis components; here we choose *w*_*Y*_ = 2. The maximum polynomial degrees *D*_ν_^max^ can be adjusted to balance the size of the basis set against fit accuracy and evaluation time; the precise parameters we choose for each molecule are given in Table S1. The basis truncation we specified here is just one, rather simple, way to obtain a finite basis. There may very well be more sophisticated methods to choose an optimal subset of the complete basis.

### 2.4 Parametrization of the Linear ACE Potentials

We define the total energy of a linear ACE model with parameters **c** corresponding to a spatial configuration of atoms (denoted by *X*, e.g., a molecule in a particular configuration) as the sum of the site energies
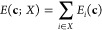
17where *E*_*i*_ is a site energy defined in eq 10. Optimal parameters are obtained by minimizing the loss function
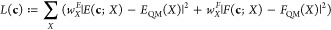
18where the *E*_QM_ and *F*_QM_ are energies and forces, respectively, in the training data, obtained from electronic structure calculations. The sum is taken over all configurations in the training set, and *w*_*X*_^*E*^ and *w*_*X*_^*F*^ are weights specifying the relative importance of energies and forces. Since the model energy and force are both linear in the free parameters, the loss can be written in a linear least-squares form
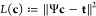
19where the vector **t** contains the QM energy and force observations and the design matrix **Ψ** contains the values and gradients of the basis evaluated at the training geometries. **Ψ** has a number of rows equal to the total number of observations (energies and force components) in the training set and a number of columns equal to the total number of basis functions.

The least-squares problem has to be regularized, especially when the basis contains high-degree polynomials.^33^ One option is to apply Tychonov regularization, where the loss function is modified as
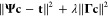
20This is widely used to regularize linear regression, often by taking **Γ** as just the identity matrix or alternatively in the case of kernel ridge regression (and Gaussian process regression) as the square root of the kernel matrix.^43^ In the present case, we use a diagonal **Γ** with entries corresponding to a rough estimate for the *p*th derivative of the basis functions
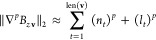
21where *n*_*t*_ and *l*_*t*_ are part of the elements of the multi-index vector **v** (cf. eq 6). This scales down high-degree basis functions, encouraging a smooth potential, which is crucial for extrapolation and is loosely analogous to the smooth Gaussian prior to GPR. The actual solutions are then found using the standard iterative LSQR solver;^44^ for the details see the Supporting Information.

In the other approach we used for solving the least-squares problem, the same **Γ** matrix is introduced but without a Tychonov term
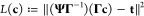
22and the solution is found using the rank revealing QR factorization^45^ (RRQR), in which we perform a QR factorization of the scaled design matrix **ΨΓ**^–1^ and truncate the small singular values below some tolerance parameter λ. For more details of the exact implementation, see refs (26 and 45). We found that when the linear system is not underdetermined, RRQR gave somewhat better solutions than LSQR. All parameters of the optimization (*w*_*X*_^*E*^, *w*_*X*_^*F*^, *p*, λ) are given in the Supporting Information.

The last modeling choice that needs to be made is the one-body term, that is, the energies of the isolated atoms of each element in our model. One can use the energy of the isolated atoms evaluated with the reference electronic structure method, which ensures the correct behavior of the model in the dissociation limit, in other words, that the force field is modeling the *binding energy* of the atoms. An alternative approach, often used in the ML fitting of molecular energies, is to take the average energy of the training set divided by the number of atoms in the molecule and assign the result to each element. In this case, the fitted model has zero mean energy. This usually improves the fit accuracy slightly, by reducing the variance of the function that we need to fit in case the data spans a narrow energy range around its average, e.g., because it came from samples of moderate temperature molecular dynamics.

A third option is to not use any reference potential energies for the fit but rather only forces. Once the coefficients are determined, the potential can be shifted by a constant energy chosen to minimize the training set energy error. In the current work, we evaluated all three strategies for ACE and found that using the isolated atom energies for the one-body term gives slightly higher RMS errors but leads to far superior extrapolation. The other two strategies (using the average energy for the one-body term and fitting only to forces) result in similar, somewhat lower test set errors but inferior physical extrapolation properties.

As mentioned in the Introduction, we view tests on data sets such as MD17 and ISO17 as *proxies*: the models thus created are not useful for any scientific purpose. The promise of ML force fields is greatest when the intention is to describe a very wide variety of compounds and conformations, perhaps including chemical reactions. With this in mind, the most natural choice for the one-body term is to choose it to match the energy of the isolated atom in vacuum. This choice is independent of any particular data set, and the apparent advantages of the other choices in terms of lower errors are expected to diminish in the limit of a large and wide ranging data set.

## 3 Results

### 3.1 MD17

The original MD17 benchmark data set consists of configurations of 10 small organic molecules in vacuum sampled from density functional theory (DFT) molecular dynamics simulations at 500 K.^38^ It has recently been recognized that some of the calculations in the original data set did not properly converge; in particular, many of the forces are noisy. A subset of the full data set was recomputed with very tight SCF convergence settings and is called the rMD17 (revised MD17) data set.^46^ We have used this new version of the data set and the five train-test splits as reported in ref (46). These revised training sets consist of 1000 configurations to avoid the problem of correlated training and test sets: when more than 1000 configurations are used from the full published trajectory, some of the test set configurations will necessarily fall between two neighboring training set data points that are separated by a much smaller time difference than the decorrelation time of the trajectory, resulting in an underestimation of the generalization error.^46^

Table 2 shows the mean absolute error (MAE) of the different force field models trained on 1000 configurations. For comparison, in Tables 2 and 3, we include a wide selection of models from the various classes of force field fitting approaches that we discussed in the Introduction (Table 1). They include ML approaches such as feed forward neural networks (ANI, GMsNN, and GMsNN), Gaussian process regression models (sGDML, FCHL, and GAP), and graph neural network based models (DimeNet, Schnet, Physnet, Cormorant, and PaiNN). The models on the left of Table 2 were trained by us (except for FCHL) using the exact train-test splits of rMD17, whereas the models on the right of the solid vertical line are from the literature and were trained on the original MD17 data set using different train-test splits. The precise details of the fitting procedures and parameters for each of the models can be found in the Supporting Information.

**Table 2:** Mean Absolute Error of MD17 Molecules^a^

		ACE	sGDML	FCHL^46^	GAP	ANI	FF	PaiNN^19^	GMsNN^20^	DimeNet^21^
aspirin	E	**6.1**	7.2	6.2	17.7	16.6	93.2	6.9	16.5	8.8
	F	17.9	31.8	20.9	44.9	40.6	260	**16.1**	29.9	21.6
azobenzene	E	3.6	4.3	**2.8**	8.5	15.9	112	–	–	–
	F	10.9	19.2	**10.8**	24.5	35.4	246	–	–	–
benzene	E	**0.04**	0.06	0.35	0.75	3.3	13.2	–	3.5	3.4
	F	**0.5**	0.8	2.6	6.0	10.0	105	–	9.1	8.1
ethanol	E	1.2	2.4	**0.9**	3.5	2.5	42.1	2.7	4.3	2.8
	F	7.3	16.0	**6.2**	18.1	13.4	208	10.0	14.3	10.0
malonaldehyde	E	1.7	3.1	**1.5**	4.8	4.6	45.9	3.9	5.2	4.5
	F	11.1	18.8	**10.3**	26.4	24.5	234	13.8	19.5	16.6
naphthalene	E	0.9	**0.8**	1.2	3.8	11.3	65.3	5.1	7.4	5.3
	F	5.1	5.4	6.5	16.5	29.2	292	**3.6**	15.6	9.3
paracetamol	E	4.0	5.0	**2.9**	8.5	11.5	93.9	–	–	–
	F	12.7	23.3	**12.3**	28.9	30.4	248	–	–	–
salicylic acid	E	**1.8**	2.1	**1.8**	5.6	9.2	68.4	4.9	8.2	5.8
	F	9.3	12.8	9.5	24.7	29.7	263	**9.1**	21.2	16.2
toluene	E	1.1	**1.0**	1.7	4.0	7.7	36.9	4.2	6.5	4.4
	F	6.5	6.3	8.8	17.8	24.3	183	**4.4**	14.7	9.4
uracil	E	1.1	1.4	**0.6**	3.0	5.1	43.3	4.5	5.2	5.0
	F	6.6	10.4	**4.2**	17.6	21.4	233	6.1	14.3	13.1
average MAE	E*	**0.12**	0.16	**0.12**	0.37	0.50	3.9	**0.33**	0.49	0.36
	F	**8.0**	12.8	8.6	22.5	24.1	227	**8.0**	17.3	13.0

a Energy (meV) and force (meV/Å) errors of different models trained on 1000 samples The models on the left were trained and tested using the same train-test splits of rMD17, whereas models on the right use MD17. The best model for each molecule (on the left and the right) is shown in bold font. The average energy MAE is calculated per atom. For ref (43) meV = 1 kcal/mol.

**Table 3:** Mean Absolute Error of Neural Network Models on the Original MD17^a^

		Cormorant^22^	Schnet^23^	Physnet^24^	GMdNN^20^	DTNN^30^
aspirin	E	**4.2**	5.2	5.2	5.6	–
	F	–	14.3	**1.7**	5.2	–
benzene	E	**1.0**	3.0	3.0	3.0	1.7
	F	–	7.4	**6.1**	**6.1**	–
ethanol	E	**1.3**	2.2	2.2	2.2	–
	F	–	2.2	**0.9**	1.7	–
malonaldehyde	E	**1.8**	3.5	3.0	3.0	8.2
	F	–	3.5	**1.3**	2.2	–
naphthalene	E	**1.3**	4.8	5.2	4.8	–
	F	–	4.8	**1.3**	3.5	–
salicylic acid	E	**2.9**	4.3	4.8	4.8	17.8
	F	–	8.2	**1.3**	3.5	–
toluene	E	**1.5**	3.9	4.3	3.9	7.8
	F	–	3.9	**1.3**	2.6	–
uracil	E	**1.0**	4.3	4.3	4.3	–
	F	–	4.8	**1.3**	1.7	–
average MAE	E*	**0.13**	0.29	0.29	0.28	–
	F	–	6.1	**1.9**	3.3	–

a MAE of energy (*E*, meV) and force (*F*, meV/Å) predictions of models recently published that were trained on 50 000 geometries of the original MD17 data set.

Of the descriptor-based models, sGDML, FCHL, and our linear ACE have the lowest MAE for some molecules. Overall, based on the per atom energy and force, the ACE model achieves the lowest errors averaged across the entire data set, improving on the state of the art for several individual molecules as well. It is interesting to note that of the neural network models, the PaiNN equivariant neural network achieves very low force errors, but its energy errors are almost three times higher compared to ACE and FCHL. In our view, the energy error is important because even though a molecular dynamics trajectory is only affected directly by the forces, the stationary probability distribution that MD is used to sample is *solely a function of the energy* through the Boltzmann weight, and so errors in predicted energy translate into errors of the stationary distribution and thus of all equilibrium observables.

The ANI model in this table refers to our reparametrization of the ANI architecture with pretraining; that is, the neural network weights were initialized from those of the published ANI-2x model.^17^ This was crucial for achieving the errors shown. When the weights are initialized randomly, the errors are higher by a factor of 2 (Table S2). The GAP model, using SOAP features to describe the atomic geometry (which are similar to ANI’s features), achieves similar errors to the ANI model with pretraining. The fact that ANI is only competitive with GAP if it is pretrained can be rationalized by the relative sample efficiency of kernel models compared to neural networks. The FCHL kernel models also use two- and three-body correlations as features, but they have been more carefully optimized for molecular systems and hence are able to achieve very low errors.^15^

The classical force field (FF) refers to a reparametrization of the GAFF functional form^2,47^ using the ForceBalance program^6,47^ and the rMD17 training set. This model gives at least an order of magnitude higher errors compared to the ML force fields. This is not a huge surprise but is nevertheless a quantitative characterization of the limitations of the fixed functional form for a situation in which the empirical force fields are designed to do well.

For completeness, in Table 3 we show the MAEs of the neural network models reported in the literature that were trained on 50 000 structures from the original MD17 trajectories. The test set errors of these models are probably underestimating the true generalization error because the large training set contains configurations that are correlated with the test set, as discussed above.^46^ It is still interesting to note that the Cormorant equivariant neural network^22^ achieves very low energy errors compared to PaiNN, even though it was trained on energy labels only, but the force errors for this model were not reported. On the other hand, the PhysNet^24^ graph neural network achieves remarkably low force errors compared to the other models. But similarly to the other equivariant graph neural network models, this comes at the expense of having close to three times larger energy errors compared to ACE and FCHL.

#### 3.1.1 Learning Curves

**Figure 2:**
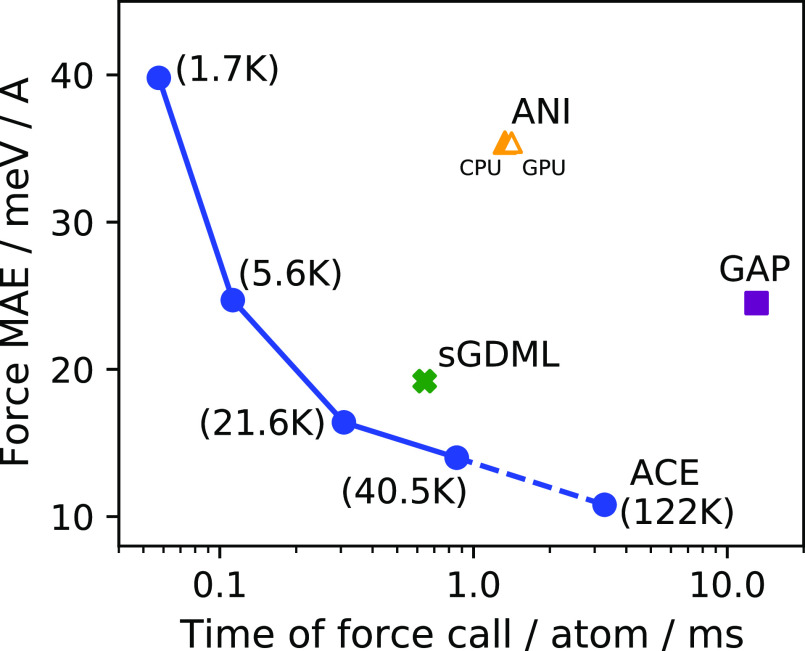
Force evaluation times. The timing of force calls per atom for the azobenzene molecule. In the case of ACE the number of basis functions is shown in parentheses. The classical force field has a timing of about 1 μs, which would not fit on this scale. For the ANI model we show both the CPU and GPU timings.

The first property to consider beyond the raw energy and force errors is the learning curve, showing how a model’s performance improves with additional training data. For kernel models such as FCHL and sGDML, the “kernel basis” grows precisely together with the training data, which is why these methods are universal approximators. Subject to the radial cutoff, the infinite set of atomic cluster expansion basis functions forms a complete basis for invariant functions, so in principle they can also be used to approximate the potential energy surface to arbitrary accuracy.^35^ In this case, however, the size of the training set and the size of the basis are decoupled. One advantage is that the evaluation cost is independent of the training set size, but we have to choose a finite basis set to work with by selecting a maximum body order and the truncation of the one-particle basis. In order to motivate our choice, we show in Figure 2 the force accuracy of ACE as a function of the basis set size and the corresponding evaluation time, trained on 1000 azobenzene configurations (the largest molecule in MD17).

The timings were obtained using a 2.3 GHz Intel Xeon Gold 5218 CPU. For context, we show the accuracy and evaluation time of the other ML models we trained, each called in their native environment: ACE in Julia, GAP via the Fortran executable, and sGDML and ANI directly from their respective Python packages. (Note that in the case of ANI considerable speed up could be achieved using a GPU when multiple molecules are evaluated simultaneously, see Figure S1, though our single-molecule results are in agreement with the timings reported in the original ANI paper^17^). The solid part of the ACE curve corresponds to four-body potentials (ν = 3), and we varied only the polynomial degrees, whereas for the last point (dashed), we increased the body order to five because the four-body part of the curve showed saturating accuracy. Increasing the body order further is likely to bring the error down even more; however, the cost of evaluation would also grow unacceptably if all basis functions for the given body and polynomial degree were retained. In the future, effective sparsification strategies need to be developed that would allow the inclusion of some high-body-order basis functions without the concomitant very large increase of the overall basis set size. For the purposes of the present paper, for each molecule in MD17 we selected a basis set size such that the evaluation cost was roughly comparable with that of the other ML models. (Note however that in a real ML force field application, one might very well choose a much smaller basis, e.g., 10K, to take advantage of the submillisecond evaluation times.)

**Figure 3:**
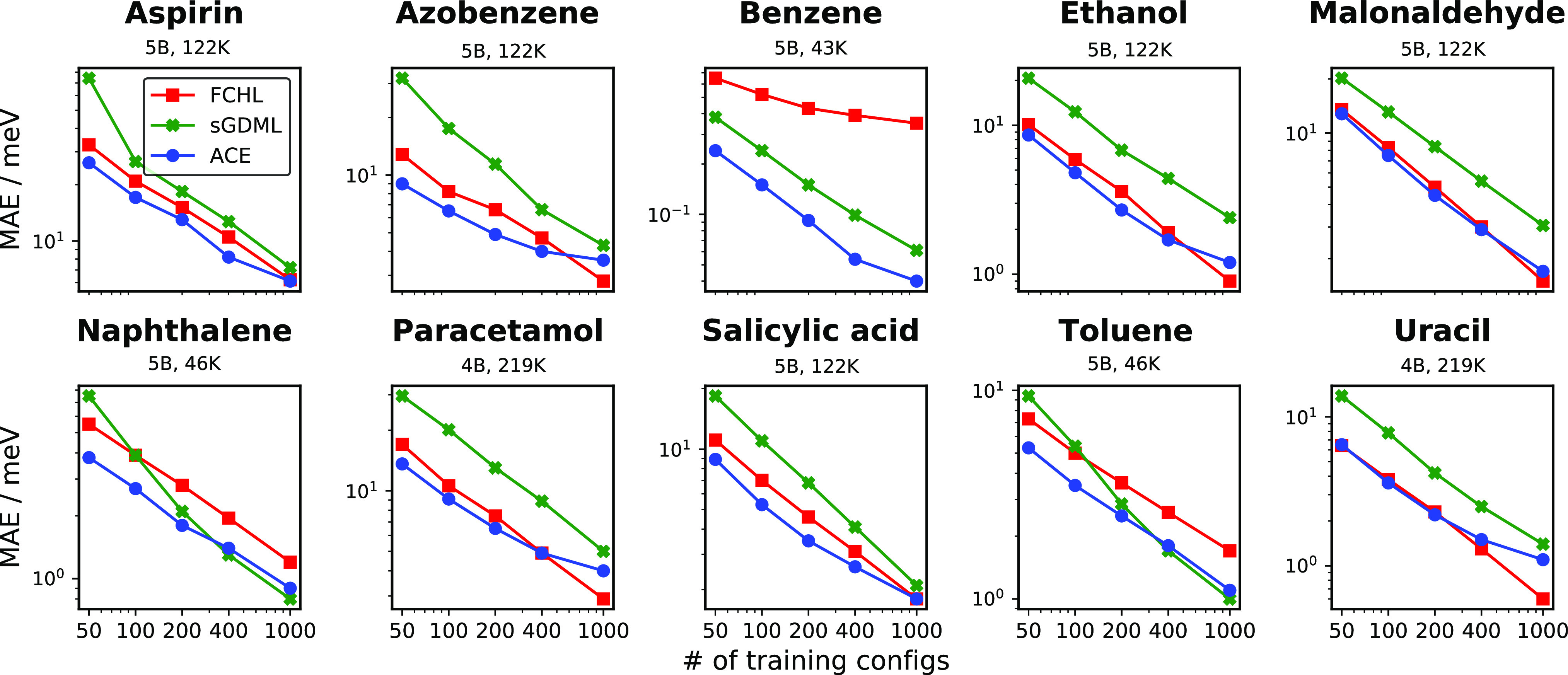
Energy learning curves. The learning curves of the best performing models on the rMD17 data set. The body order and basis set size for the ACE models are given under the title of each panel.

In Figure 3 we show the learning curves for linear ACE and sGDML (the best models we trained from Table 2) and compare them to the literature results of FCHL.^46^ The low-body-order linear ACE is equal to or better than the other many-body kernel models in the low-data limit, but with additional training data the kernel models overtake ACE in several cases. The latter also saturates, showing the limitations of the relatively low-body-order model. The learning curves for the forces are given in Figure S2 and show a broadly similar trend, with less pronounced saturation for ACE.

#### 3.1.2 Normal-Mode Analysis

The normal modes and their corresponding vibrational frequencies characterize the potential energy surface near equilibrium. This is interesting in the context of the MD17 models because their training set contains geometries sampled at 500 K which means they are, in general, far from the equilibrium geometry. The ability of the models to describe the minima of the PES, even if it is not in the training set, is particularly important when considering larger systems with potentially many local minima, where finding all the different local minima at the target level of theory can be infeasible.

To test how well the different models infer the normal modes, we took the DFT-optimized geometry of each of the 10 molecules and rerelaxed them with the force field models. At the force field minima we carried out a vibrational analysis to find the normal modes and their corresponding vibrational frequencies.

**Figure 4:**
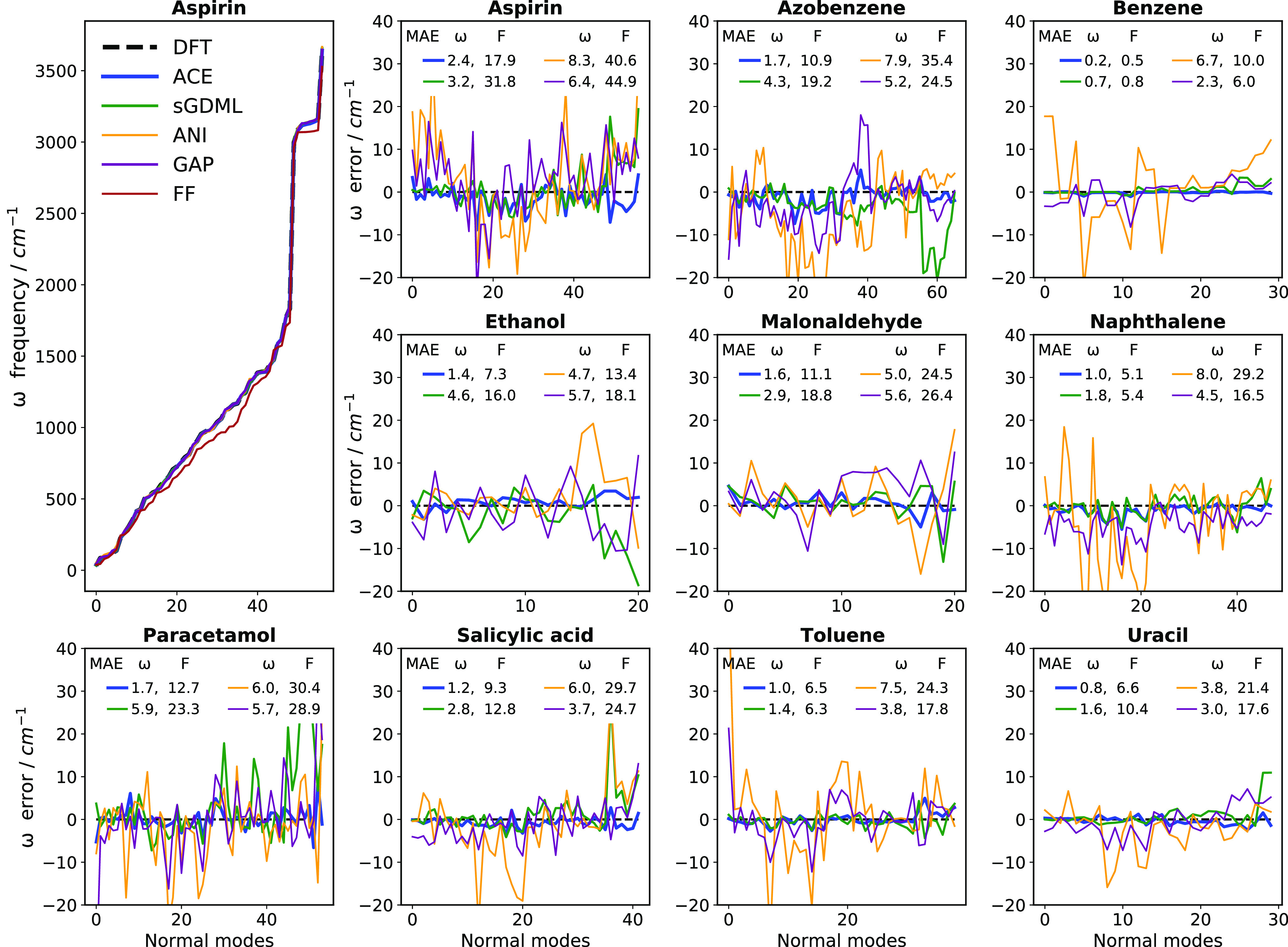
Normal-mode frequency test. The frequency error of the normal modes of each of the MD17 molecules. The legend shows the frequency (ω) MAE (in cm^–1^) and also the force (*F*) MAE (in meV/Å) from Table 2 for each model.

Figure 4 shows the errors in the predicted normal-mode vibrational frequencies for each of the 10 MD17 molecules. The ACE model achieves the lowest error for all 10 molecules, surprisingly even for those for which sGDML has lower errors based on the 500 K MD test set of Table 2. For example, for toluene sGDML has both lower energy and force errors, but at the same time the ACE model has significantly lower errors in predicting the vibrational frequencies, achieving a MAE of 1.0 cm^–1^ compared to sGDML with an error of 1.4 cm^–1^. Upon observing the individual molecules in Figure 4, it is notable that the ACE model has the lowest fluctuation in the errors of the normal modes, achieving nearly uniform accuracy across the entire spectrum. The case of benzene also shows the limitations of characterizing the models by the force MAE alone. The linear ACE model has only slightly lower force MAE than sGDML (0.5 meV/Å compared to 0.8 meV/Å), but the normal-mode frequency prediction is more than three times more accurate: 0.2 cm^–1^ compared to 0.7 cm^–1^. The linear ACE model has very low errors for all normal modes, whereas sGDML has much higher errors for the high-frequency modes.

Similarly, in the case of aspirin, even though the ANI model has lower MAE on the test set for both energies and forces than the GAP model, its vibrational frequency error is significantly larger than that of GAP (8.3 cm^–1^ compared to 6.4 cm^–1^). We also compared the models to the accuracy of a classical force field. The normal-mode frequency errors of the empirical FF are about 10 times higher than the errors of the ML force fields. These errors do not fit on the scale of Figure 4 but are reported in Figure S3.

#### 3.1.3 Extrapolation in Temperature

When building a new force field for a molecule, beyond high accuracy, we also need robustness, by which we mean that there should not be areas of accessible configuration space where the model predictions are unphysical or nonsensical. Sometimes called “holes” in the potential energy surface, these can be remedied by regularization^33^ or by iterative fitting^39^ and additional data.^48^ In the context of the MD17 benchmark, with its fixed training set, we test the robustness of the models we fitted by running short molecular dynamics (MD) simulations with each model. Separate MD simulations were run at several temperatures between 350 and 950 K using a Langevin thermostat and a time step of 0.3 fs. (Higher temperatures were not considered because most organic molecules undergo thermal decomposition at temperatures above 1000 K.) Five independent MD runs were initialized starting from different configurations. After equilibrating for 500 steps, 10 samples were taken 200 time steps apart from each of the trajectories. These constitute the new test set specific for each molecule, model, and temperature.

**Figure 5:**
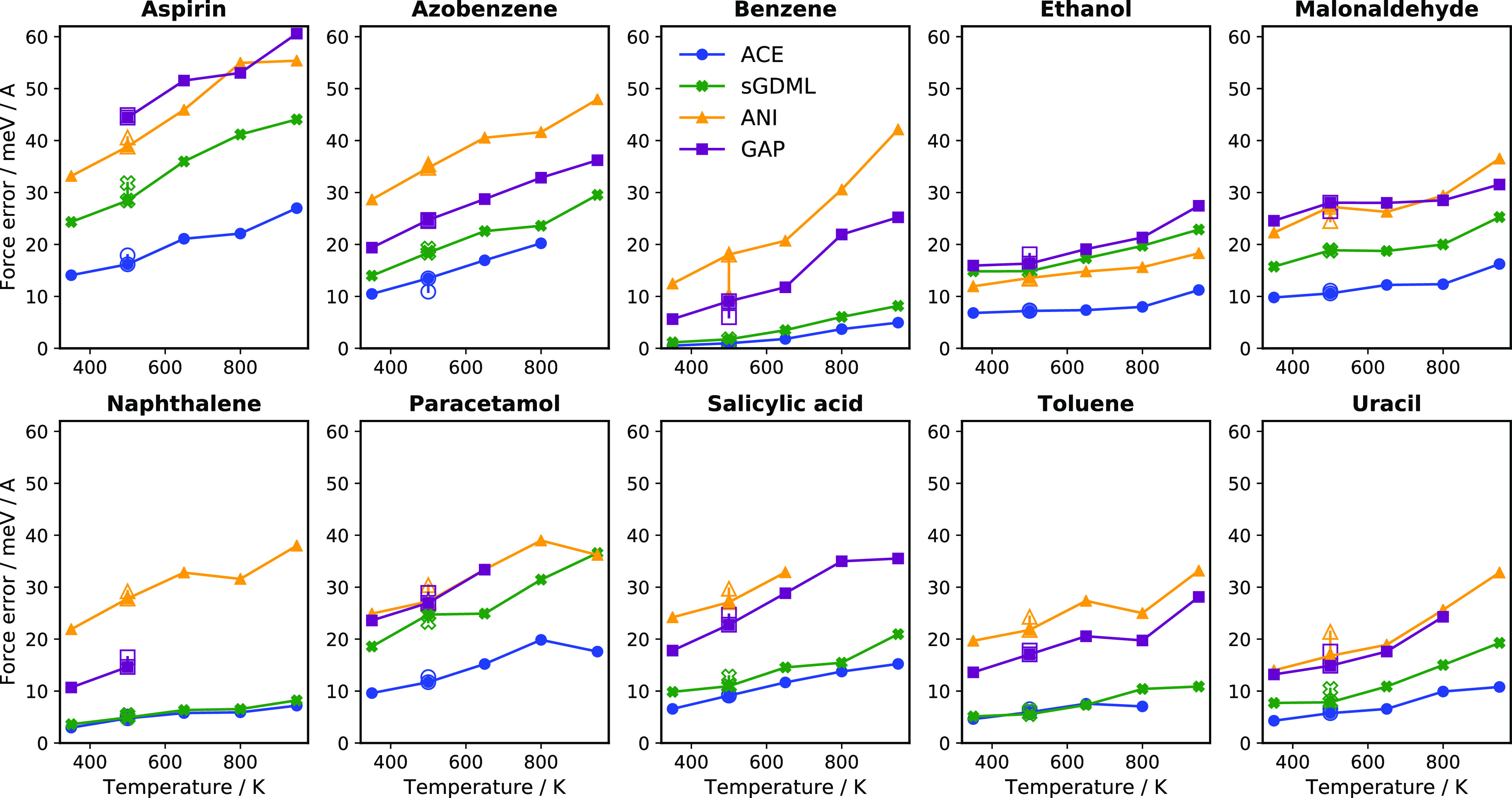
Extrapolation force error. The mean absolute force error as a function of temperature sampled from five independent MD trajectories driven by each model, at several temperatures. The empty marker corresponds to the MAE on the 500 K test set of Table 2.

The energies and forces of the new test configurations were recomputed with DFT to estimate the accuracies of the different models at each temperature along their own MD trajectories. The force errors are shown in Figure 5 while the energy errors showing the same trends are given in Figure S6. Where a point is missing, the model hit a hole in the potential, and the MD run was terminated. This happened most often with the GAP model, indicating that this potential was the least regular. The linear ACE and ANI models can also be prone to hitting holes in the potential at the highest temperatures. Of all models sGDML was the most stable; it always kept the molecule intact even at 950 K for the duration of the simulations. Such extreme stability is not necessarily chemically realistic (see the next section on extrapolation to bond breaking).

Looking at the increase in errors with temperature for the different models, we can see that the linear ACE often keeps the errors low with a small slope whereas the other models show a clearer increase as the temperature increases. This can be best observed for ethanol, malonaldehyde, and uracil. It is notable that the model that works best at lower temperatures (in the training regime) also works best at higher temperatures confirming that the models are able to smoothly extrapolate away from the training data. Furthermore, we can see a good agreement of the test set force MAE in Table 2 with the force MAEs estimated from the models’ own trajectories. This hints that the models explore similar regions of the configuration space as the original *ab initio* trajectories.

#### 3.1.4 Extrapolation Far from the Training Set

To test the extrapolation properties of the different models further, we looked at two tests probing the torsional profile of azobenzene and the O–H bond breaking in ethanol. Both of these tests probe how far away the models can smoothly extrapolate from the training data.

We carried out these tests with several different versions of the linear ACE models differing in the definition of their one-body terms because we expect this choice to make a significant difference in how chemically reasonable the fitted models are far from the training set. We denote the ACE models fitted using force data only by ACE F. This has the lowest force error on the test set (comparison shown in Table S3). For the other two ACE models, energies were also included in the training. They differ in the one-body term only; the model using average per-atom training set energy is denoted as ACE AVG, whereas the model using the isolated atom energies as the one-body term is denoted ACE E0. The third option is the natural choice, as this ensures that if all atoms are separated from each other the predicted energy will correctly correspond to the sum of the isolated atom energies.

**Figure 6:**
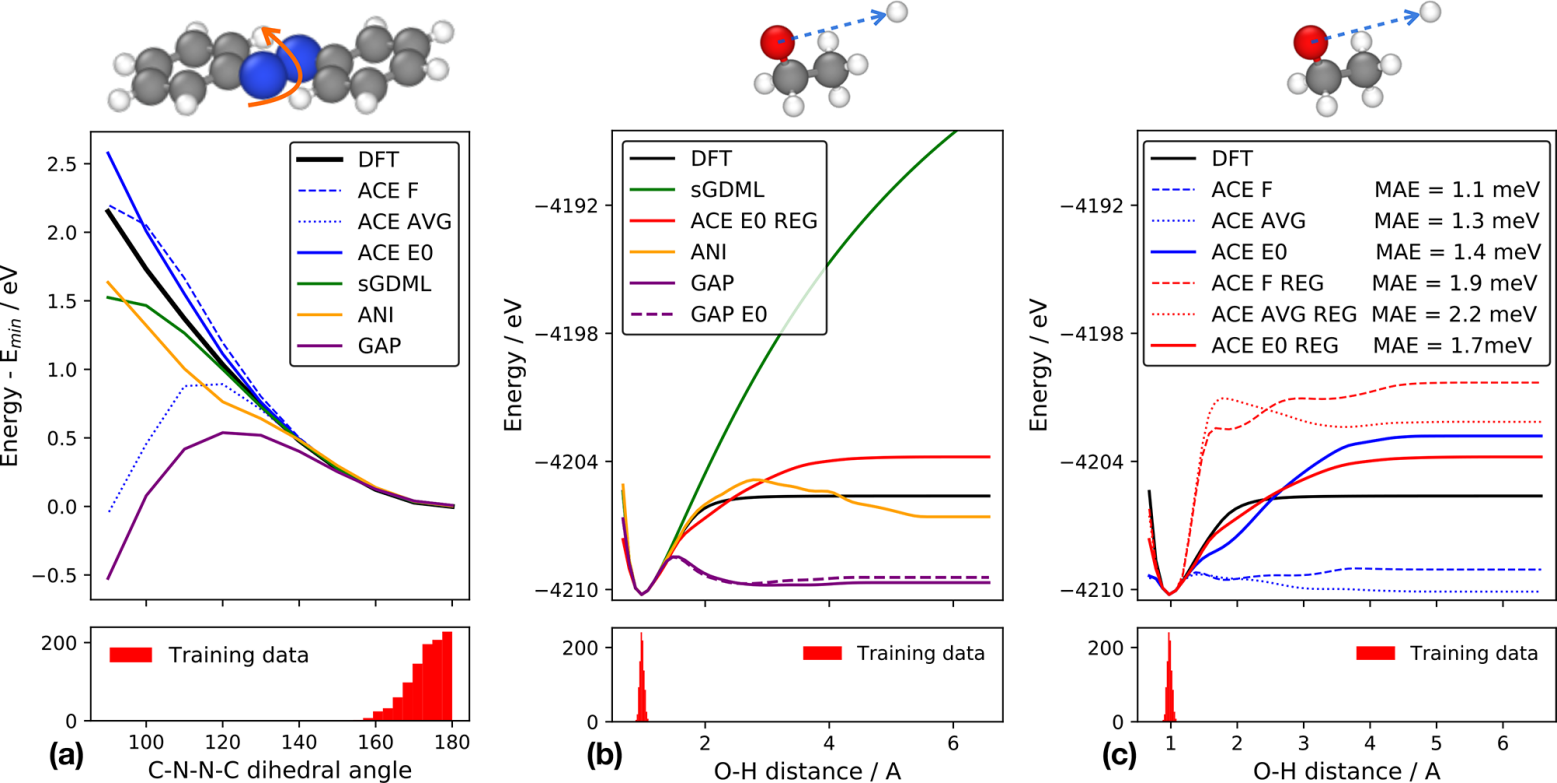
Extrapolation test far away from training data. The bottom panels show the histogram of the variables in the training set. (a) The energy change predicted by the different models as the C–N–N–C dihedral angle of azobenzene is decreased from the equilibrium 180°. ACE F refers to training on forces only, ACE AVG refers to using average per atom energy as the one-body term, and ACE E0 refers to using the isolated atom energy as the one-body term. (b) Change in energy as the O–H bond distance of ethanol is extended from equilibrium, as predicted by the different models. (c) Comparison of ACE models (i) with lowest force MAE and (ii) with slightly stronger regularization, the latter indicated with the REG label.

Figure 6a shows the torsional energy profile of the azobenzene molecule. The ACE E0 model with the isolated atom one-body term is able to extrapolate furthest, somewhat overestimating the energy, while the ANI and sGDML models also extrapolate smoothly but slightly underestimate the energy. The linear ACE model with the average energy one-body term and the GAP model fail to extrapolate and predict a completely nonphysical drop in energy for smaller values of the dihedral angle.

Figure 6b shows the energy profile as the O–H distance is varied starting from the equilibrium geometry of ethanol. The only force field that shows qualitative agreement with DFT is the ACE E0 model. (Note that we do not expect any of the fitted models to quantitatively reproduce the DFT energy profile, even when the isolated H atom is described correctly by design, because the C_2_H_5_O^•^ radical is not.) We attribute this success to the explicit body-ordered nature of the linear ACE model, including using the isolated atom as the one-body term, and careful regularization—as was the case in a similar test for other polynomial models.^26^Figure 6c shows a detailed comparison of the different ACE models together with their test set MAE value. This shows that having the lowest possible test set error does not coincide with the most physically reasonable model, and using stronger regularization can lead to much smoother extrapolation. The more strongly regularized ACE models with relatively higher force error are still significantly more accurate than sGDML, ANI, GAP, or the classical force field.

Interestingly, having the isolated atom as the one-body term is not sufficient for good extrapolation. This is shown by the two different GAP models in Figure 6b, which show essentially no difference from the extrapolation, presumably due to the very poor description of the radical. GAP is not an explicitly body-ordered model.

### 3.2 Fitting Multiple Molecules

Apart from sGDML, whose descriptor is tied to a given molecule with fixed topology, the models under consideration can all be fitted to multiple molecules simultaneously. Therefore, having evaluated their capacity to approximate individual potential energy surfaces one by one, it is interesting to see how the they cope with describing all of the rMD17 data set pooled together.

Table 4 shows the energy and force errors for the combined fit with linear ACE, GAP, ANI, and the empirical force field. GAP and ANI errors only go up by around 30%, reflecting the fact that these are very flexible functional forms. The ANI model (which is pretrained by starting from ANI-2x neural network weights) is now distinctly better than GAP. The empirical force field error increases by even less. In this case that is due to the use of atom types, which help to separate the energy contribution of different functional groups. The increase in the error is largest for ACE, about a factor of 2, although for most molecules it is still the combined ACE model that has the lowest error among these models.

**Table 4:** Combined Fitting of rMD17: Mean Absolute Error of the Energies (*E*, meV) and Forces (*F*, meV/Å) of Different Models When Fitting All 10 MD17 Molecules Together^a^

		ACE	GAP	ANI	FF	ANI-2x
aspirin	E	**9.9**	19.7	13.8	105.7	44.7
	F	**27.0**	46.2	35.3	287.3	76.6
azobenzene	E	**5.7**	10.8	12.9	115.1	144.4
	F	**16.6**	30.1	30.1	243.5	115.5
benzene	E	**0.8**	1.6	2.4	16.9	10.4
	F	**3.5**	9.3	10.6	125.6	24.4
ethanol	E	4.6	9.2	**2.8**	42.4	22.1
	F	21.5	35.7	**15.1**	220.6	29.0
malonaldehyde	E	5.1	9.6	**4.6**	48.8	24.2
	F	**24.1**	46.2	24.6	278.5	58.9
naphthalene	E	**3.5**	8.3	9.5	72.8	19.5
	F	**13.3**	30.8	26.2	306.7	54.1
paracetamol	E	**6.5**	12.5	10.7	102.9	29.7
	F	**20.8**	37.8	29.5	275.1	60.2
salycilic acid	E	**4.6**	9.6	7.2	83.3	21.3
	F	**18.9**	35.9	26.1	310.6	68.7
toluene	E	**3.5**	7.7	6.9	37.1	20.1
	F	**13.1**	29.7	23.3	184.2	42.5
uracil	E	**2.8**	5.1	4.5	50.6	14.8
	F	**15.6**	26.1	21.9	265.3	48.9
avg MAE	E*	**0.31**	0.62	0.46	4.2	2.1
	F	**17.4**	32.8	24.3	249.7	57.9

a The average energy error (E*) is calculated on a per-atom basis.

In addition, we also show the performance of the original unmodified ANI-2x model (its energies and forces were tested against values recomputed with exactly the same electronic structure method and parameters that were used in its fitting^49^). Its energies and forces are better than those of the empirical force fields by factors of around 2–3 and 5, respectively. (The exception is azobenene, for which its energies are worse.) The difference between ANI-2x and the retrained ANI is about a factor of 2–4 for energies (the average over all the molecules is at the high end) and a factor of 2 for forces.

The other commonly used benchmark data set for machine-learning-based molecular force fields that contains multiple molecules is ISO17.^23^ The full data set contains 5000-step *ab initio* molecular dynamics simulation trajectories of 129 molecules, all with the same chemical formula C_7_H_10_O_2_. The standard task is to train a force field using 4000 randomly selected configurations of 103 molecules (so about 400K configurations altogether, although these are highly correlated) and evaluate them on the remaining 1000 structures of the trajectory (“known molecules”) and on the full trajectories of the “unknown molecules”. We note that when all 400K training configurations are used, the conformations of known molecules that are usually reported as a test set are very close to the training set, at most one or two MD steps away on the trajectory from the actual training set, so the error measured on these is essentially the same as the training error.

We trained a linear ACE model on only a total of 5 000 configurations and a GAP model on only a total of 10 000 configurations sampled uniformly from the training set and evaluated them on both the known and unknown molecules. The results in Table 5 show that the linear ACE model performs significantly better than GAP, achieving errors in the same ballpark as the other methods for the unknown molecules but using orders of magnitude less training data. In particular, the ACE model matches the energy error of the state of the art GM-sNN^20^ on the unknown molecules, demonstrating its excellent extrapolation capabilities. For all the neural network models, the error on known molecules is quite a bit lower than that for the unknown molecules, which we consider to be a sign of overfitting. For ACE and GAP, the error is still lower but by a much smaller factor, helped by the explicit regularization. Tellingly, the most similar ratio is for GM-sNN, which is a shallow neural network.

**Table 5:** ISO17 Test: Mean Absolute Error in Energies (*E*) and Forces (*F*) of Models Trained on ISO17^a^

		ACE	GAP	Schnet	Physnet	GM-sNN	GM-dNN
known molecules	E	16	54	16	4	17	7
	F	43	102	43	5	28	12
unknown molecules	E	**85**	169	104	127	**85**	118
	F	75	128	95	**60**	72	85

a The results on the “known molecules” are essentially training errors (see text). The bold indicates the lowest error. The linear ACE is trained on 5 000 configurations, GAP on 10 000 configurations, and the neural networks on 400K (quite correlated) configurations.

### 3.3 Flexible Molecule Test: 3BPA

**Figure 7:**
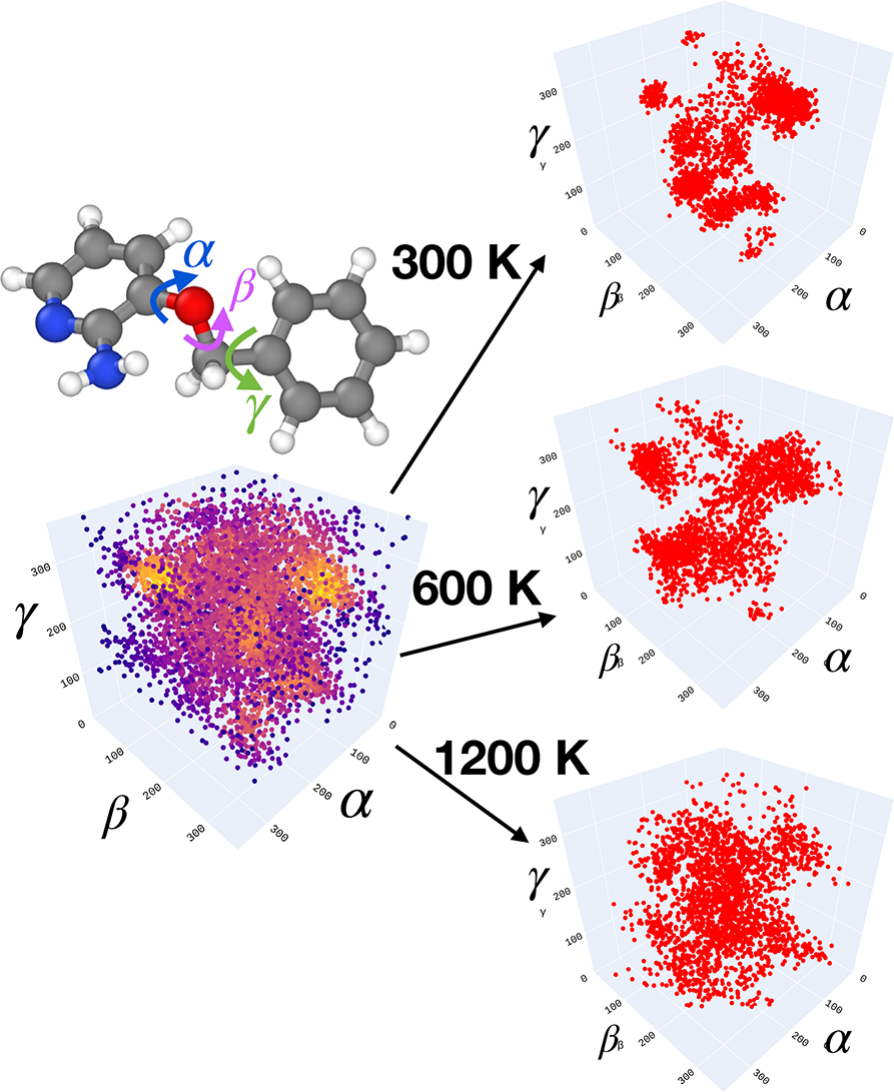
3BPA data set. The three freely rotating angles of the 3BPA molecule together with a characterization of the three different data sets sampled at different temperatures showing how the phase space sample increases significantly with temperature.

Finally, noting that all the MD17 molecules are rather rigid, our last test is to assess the capabilities of the different force field models on a more challenging system that has relevance for medicinal chemistry applications. We created a new benchmark data set for the flexible druglike molecule 3-(benzyloxy)pyridin-2-amine (3BPA).^50^ Though smaller than typical druglike molecules, with a molecular weight of 200, this molecule has three consecutive rotatable bonds, as shown in Figure 7. This leads to a complex dihedral potential energy surface with many local minima, which can be challenging to approximate using classical or ML force fields.^51^

#### 3.3.1 Preparation of the Data Set

To prepare a suitable training data set, we started by creating a grid of the three dihedral angles (α, β, and γ) removing only the configurations with atom overlap. From each of the configurations corresponding to the grid points, we started short (0.5 ps) MD simulations using the ANI-1x force field.^28^ This time scale is sufficient to perturb the structures toward lower potential energies but is not enough to significantly equilibrate them. In this way we obtained a set of 7000 configurations as shown in the left panel of Figure 7. From the distribution of dihedral angles, five different densely populated pockets were identified in the space of the three dihedral angles. One random configuration was selected from each of the five pockets, and a long 25 ps MD simulation was performed at three different temperatures (300, 600, and 1200 K) using the Langevin thermostat and a 1 fs time step. We sampled 460 configurations from each of the trajectories starting after a delay of 2 ps. In this way the final data set of 2300 configurations was obtained. The configurations were re-evaluated using ORCA^52^ at the DFT level of theory using the ωB97X exchange correlation functional^53^ and the 6-31G(d) basis set. (These settings are similar to that used in the creation of the ANI-1x data set.^49^) From the total data set we created two training sets, one using 500 randomly selected geometries from the 300 K set and another one labeled “mixed-T”, selecting 133 random configurations from each of the trajectories at the three temperatures. The rest of the data in each case makes up the three test sets, each corresponding to a different temperature. The right-hand panels of Figure 7 show the distribution of dihedral angles in the test sets. At 300 K the separate pockets of the configuration space are sampled mostly individually, whereas at 1200 K the distribution widens significantly, and the sampling connects the pockets across multiple barriers with ease.

#### 3.3.2 Comparison of Force Fields Models

We trained linear ACE, sGDML, ANI, and GAP force fields and reparametrized the bonded terms of a classical force field (FF) using the 300 K and the mixed-T training sets. Table 6 shows the energy and force RMSEs of the different models alongside the general purpose ANI-2x force field errors on the same configurations. Just as before, the weights of the retrained ANI model were initialized from the ANI-2x weights, giving it a considerable advantage over the other models, especially because the DFT functional and basis set that we use are the same as those of the underlying DFT method of the ANI-2x model.

**Table 6:** Root-Mean-Squared Error of the Energy (meV) and Force (meV/Å) Predictions of Different Models of the Flexible 3BPA Molecule

		ACE	sGDML	GAP	FF	ANI	ANI-2x
fit to 300 K							
300 K	E	**7.1**	9.1	22.8	60.8	23.5	38.6
	F	**27.1**	46.2	87.3	302.8	42.8	84.4
600 K	E	**24.0**	484.8	61.4	136.8	37.8	54.5
	F	**64.3**	439.2	151.9	407.9	71.7	102.8
1200 K	E	85.3	774.5	166.8	325.5	**76.8**	88.8
	F	187.0	711.1	305.5	670.9	**129.6**	139.6
fit to mixed-T							
300 K	E	**9.3**	11.7	27.7	86.0	21.6	38.6
	F	**30.5**	55.1	85.7	307.8	56.4	84.4
600 K	E	**19.7**	25.6	50.2	115.6	40.3	54.5
	F	**54.4**	94.3	123.8	392.0	81.4	102.8
1200 K	E	**47.1**	78.2	103.0	268.8	77.6	88.8
	F	**118.4**	177.1	217.8	634.3	131.0	139.6

For the case of training on the 300 K configurations, the linear ACE and sGDML models are able to achieve very low errors when tested at the same temperature, but the ACE model shows significantly better extrapolation properties for the configurations sampled at higher temperatures. The model extrapolating most accurately to 1200 K is the retrained ANI force field, but the linear ACE is not far behind, especially considering how poor the extrapolations of the other models are. Just as for the smaller molecules, the fitted empirical force field shows much higher errors, about a factor of 2–4 for energies and a factor of 4 for forces compared with the ANI-2x force field. Only at 1200 K does ANI-2x become competitive with the ACE trained at 300 K.

Training on the mixed-T training set leads to a significant drop in the errors at the higher temperature test sets for all ML models but not for the empirical force field. The linear ACE model achieves the lowest error in every case, showing an approximately 40% decrease in the error for the high-temperature test set. The other ML models improve also, by even bigger factors (because their extrapolation power was less). The gains over the general ANI-2x force field, nearly a factor of 2 in energy for all three test sets, show the potential scope for parametrizing such custom force fields in medicinal chemistry applications. The errors in the empirical force field are mostly unchanged, quantifying the limitations of the fixed functional form when describing the anharmonic high-energy parts of the potential energy surface.

**Figure 8:**
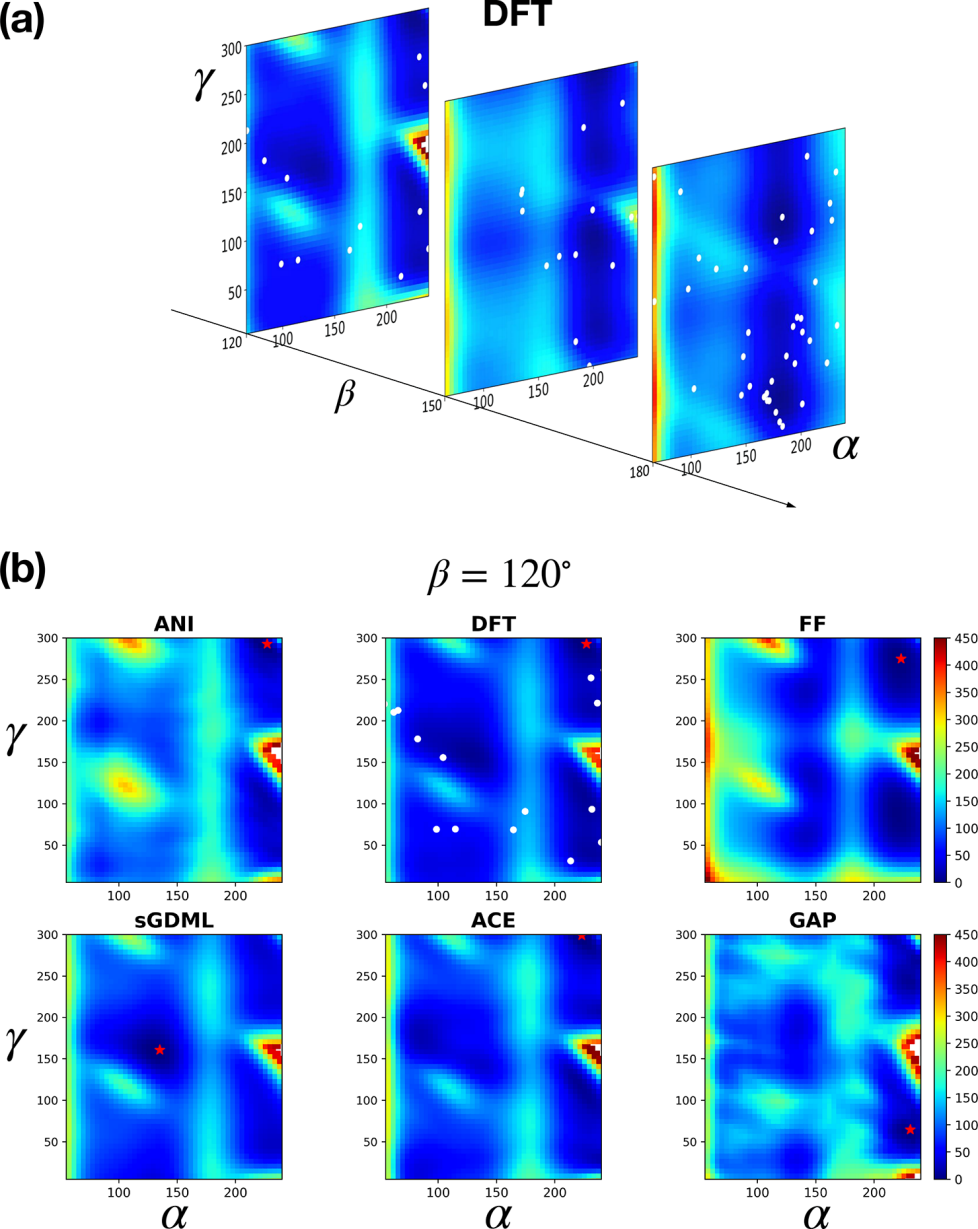
Dihedral PES of 3BPA. (a) The dihedral potential energy landscape of 3BPA for different fixed β, as predicted by DFT. (b) The β = 120° section of the PES for the different force field models. The white dots on the DFT PES correspond to configurations from the training set that lie within ±10^*o*^ of the planes considered here, and the red star shows the position of the energy minimum on each slice.

To look beyond the energy and force RMSE, we performed a constrained geometry optimization using the different force field models and DFT to map out the dihedral potential energy surface of the molecule. The complex energy landscape is visualized in Figure 8a at three different fixed values of β, in the α–γ plane, limiting the range to avoid overlapping atoms. Figure 8b shows a comparison of the ML and empirical force fields with DFT for the case of β = 120°, the plane with the fewest training data points. Analogous results for the other two values of β are reported in Figures S7 and S8. The energy landscape of the empirical force field has most of the features of the DFT landscape and is even correctly predicting the position of the lowest energy minimum in the β = 120° plane. Some of the potential energies on this plane are clearly too high however. On the other hand, the landscape of the GAP model is quite irregular; some of the most basic features are either missing or blurred together. The ANI landscape is also quite irregular, somewhat less than GAP, and some of the high-energy peaks are too high and too broad. This is an example where the fixed functional form of the classical force field gives better extrapolation behavior to parts of the configuration space where there is little training data. The RMSE results clearly do not give a full, perhaps not even a very useful, distinction between these models.

The ACE and sGDML models reproduce the landscape much more closely (and indeed these are the models with the lowest RMSE as well). Some differences include the sGDML getting the position of the lowest energy minimum wrong and ACE having too high a peak at α = 230°, γ = 150°.

## 4 Conclusions

In this paper we have demonstrated how the atomic cluster expansion framework can be used as linear models of molecular force fields. We showed that body-ordered linear models built using the ACE basis are competitive with the state of the art short-range ML models on a variety of standard tests. Furthermore, we carried out a number of “beyond RMSE” tests to compare the ML approaches and to study the smoothness and extrapolation properties of the fitted force fields: vibrational frequencies, force-field-driven molecular dynamics, and extrapolation to bond-breaking.

We also introduced a data set on a flexible druglike molecule, with the idea that testing the performance on it is more predictive of the quality of the model for medicinal chemistry applications. The linear ACE model was significantly smoother than other transferable models and was able to extrapolate to higher potential energy regions than all other models.

We showed that the ACE framework allows us to build accurate force fields with very low evaluation cost. Together with competing approaches that are in the recent literature and in our comparison tables, the prospects are good for being able to carry out large-scale simulations of systems ranging from biomolecular applications to other complex molecular systems such as polymers with electronic structure accuracy in the near future. A number of bottlenecks remain for ACE, which include the steep increase in the number of basis functions as new chemical elements are added to the model. This can be tackled via sparsification strategies, which is the focus of our future work. Furthermore, the inclusion of long-range electrostatics and charge transfer is essential for the simulation of biomolecular systems, and an integration of these into the ACE framework is also underway. Currently ACE is implemented in the Julia language but can readily be called from Python via the atomic simulation environment (ASE). The fitted models can also be evaluated via LAMMPS.

## Supporting Information Available

Parameters of all the ML and empirical force field fits, a comparison of pretrained and randomly initialized ANI models, illustration of the different CPU and GPU scaling for ANI and MD17 learning curves for the forces, normal-mode vector analysis of the MD17 models, energy errors of the MD runs with the different models, and a comparison of different one-body energy ACE models and energy landscapes for 3BPA with β = 150° and β = 180° (PDF)
3BPA data set (ZIP)
