# Supplementary material for: Linear Atomic Cluster Expansion Force Fields for Organic Molecules: Beyond RMSE
Source: J Chem Theory Comput. 2021 Nov 4;17(12):7696–711. doi: 10.1021/acs.jctc.1c00647 (PMC8675139; doi:10.1021/acs.jctc.1c00647)
Supplement: Supplementary file 1 — ct1c00647_si_001.pdf [file ct1c00647_si_001.pdf]

Supporting Information for Linear Atomic Cluster Expansion Force Fields for Organic Molecules: beyond RMSE

Dávid Péter Kovács,*,† Cas van der Oord,† Jiri Kucera,† Alice E. A. Allen,‡ Daniel J. Cole,¶ Christoph Ortner,§ and Gábor Csányi†

†*Engineering Laboratory, University of Cambridge, Cambridge, CB2 1PZ UK*

‡*Department of Physics and Materials Science, University of Luxembourg, L-1511 Luxembourg City, Luxembourg*

¶*School of Natural and Environmental Sciences, Newcastle University, Newcastle upon Tyne NE1 7RU, United Kingdom*

§*Department of Mathematics, University of British Columbia, Vancouver, BC, Canada V6T 1Z2*

E-mail: dpk25@cam.ac.uk

1 MD17

1.1 Details of the fits

1.1.1 ACE fits

The ACE fits used 0.77 Å inner and 4.4 Å outer cut-off for the many-body part and 5.5 Å outer cutoff for the longer range pair potential fitted together with the ACE. The only exception was naphthalene where the outer cutoffs were doubled to account for the longer range effects of the extended conjugated system. The loss function used had a weight 30 on the energies and 1 on the forces when fitting to both in both the isolated atom One-body and the average energy One-body case.

The regularized linear least squares problem was solved either by rank revealing QR factorisation or by the iterative LSQR algorithm. To be able to use the LSQR algorithm we have to rewrite eq (20) in the scaled coordinates $\Gamma\mathbf{c}$ by rescaling the design matrix as

$$L(\mathbf{c}) := \|\left(\Psi\Gamma^{-1}\right)\left(\Gamma\mathbf{c}\right) - \mathbf{t}\|^2 + \lambda\|\Gamma\mathbf{c}\|^2 \tag{1}$$

Writing the problem in this form allowed us to use the standard implementation of the algorithm in the `IterativeSolvers.jl` package.

The exact parameters used for each of the MD17 fits are shown in Table S1, where λ denotes the weight on the ridge penalty for LSQR or the tolerance parameter for RRQR.

Table S1: **Table of ACE fit parameters**

	**Fit to**	$\mathbf{D_\nu^{max}}$	**Regularization**	**p**	λ	**Norm**	**E_MAE**	**F_MAE**
	E + F	20, 20, 20, 8	ITLSQ	1.5	0.25	4.4	6.1	19.1
**Aspirin**	F only			1.2	0.15	8.7	6.1	17.9
	Avg E0			1.3	0.15	1.6	5.9	18.7
	E + F	19, 19, 19, 0	RRQR	1.7	5e-8	8.2	3.4	12.8
**Azobenzene**	F only		ITLSQ	1.3	0.1	2.2	3.5	10.9
	Avg E0		RRQR	1.9	1e-8	96	3.5	14.8
	E + F	17, 17, 17, 17	RRQR	0.9	1e-8	15.8	0.04	0.5
**Benzene**	F only			1.6	1e-7	16.9	0.04	0.5
	Avg E0			0.7	1e-8	15.9	0.04	0.5
	E +F	20, 20, 20, 8	ITLSQ	1.1	0.25	1.8	1.4	8.4
**Ethanol**	F only			1.3	0.05	1.4	1.2	7.3
	Avg E0			1.2	0.15	0.9	1.4	8.2
	E +F	20, 20, 20, 8	ITLSQ	1.35	0.1	3.5	1.9	12.0
**Malonaldehyde**	F only			1.3	0.05	2.0	1.7	11.1
	Avg E0			1.1	0.15	1.5	1.8	11.5
	E +F	20, 20, 20, 16	RRQR	1.9	2e-8	13.2	0.9	5.1
**Naphthalene**	F only			2.4	8e-8	17.4	0.9	5.1
	Avg E0			1.6	1e-8	16.2	0.9	5.2
	E +F	18, 18, 18, 0	ITLSQ	1.0	0.1	3.5	4.0	14.9
**Paracetamol**	F only			1.5	0.05	7.0	4.0	12.7
	Avg E0			1.2	0.2	1.7	3.8	13.8
	E +F	20, 20, 20, 8	ITLSQ	1.3	0.2	3.1	2.3	11.2
**Salicylic acid**	F only			1.6	0.05	2.1	1.8	9.3
	Avg E0			1.1	0.25	1.2	2.2	10.5
	E +F	20, 20, 20, 16	RRQR	0.9	2e-7	7.5	1.1	6.7
**Toluene**	F only			2.0	1e-6	8.6	1.1	6.5
	Avg E0			1.9	1e-8	10.7	1.1	6.7
	E +F	18, 18, 18, 0	ITLSQ	1.3	0.05	4.1	1.4	8.7
**Uracil**	F only			1.3	0.05	2.7	1.1	6.6
	Avg E0			0.9	0.15	2.0	1.2	7.8

1.1.2 ANI training

The ANI models were trained using the Torchani framework.[1] For the learning we followed the tutorial in the Documentation using the default parameters for the cutoffs and the optimization of the weights. We trained two versions of the potential for each molecule, one where the weights were initialized randomly, and another one where we applied pre-training by starting from the weights of the ANI-2x model. A comparison of the mean absolute errors is shown in Table S2. The pre-trained model achieves much lower errors in every case. We included the pre-trained ANI only in the comparison table in the main manuscript. It is important to note though, that comparing to the randomly initialised model would be more fair, as the other models were all trained from scratch.

Table S2: **Pre-trained and randomly initialized ANI models.** Mean Absolute Error of the energy (meV) and force (meV / Å) predictions of the pre-trained and randomly initialized ANI models.

		**ANI-pre**	**ANI-rand**
**Aspirin**	Energy	16.6	25.4
	Force	40.6	75.0
**Azobenzene**	Energy	15.9	19.0
	Force	35.4	52.1
**Benzene**	Energy	3.3	3.4
	Force	10.0	17.4
**Ethanol**	Energy	2.5	7.7
	Force	13.4	45.6
**Malonaldehyde**	Energy	4.6	9.4
	Force	24.5	52.4
**Naphthalene**	Energy	11.3	16.0
	Force	29.2	52.2
**Paracetamol**	Energy	11.5	18.2
	Force	30.4	63.3
**Salicylic acid**	Energy	9.2	13.5
	Force	29.7	53.0
**Toluene**	Energy	7.7	12.6
	Force	24.3	52.9
**Uracil**	Energy	5.1	8.3
	Force	21.4	44.1

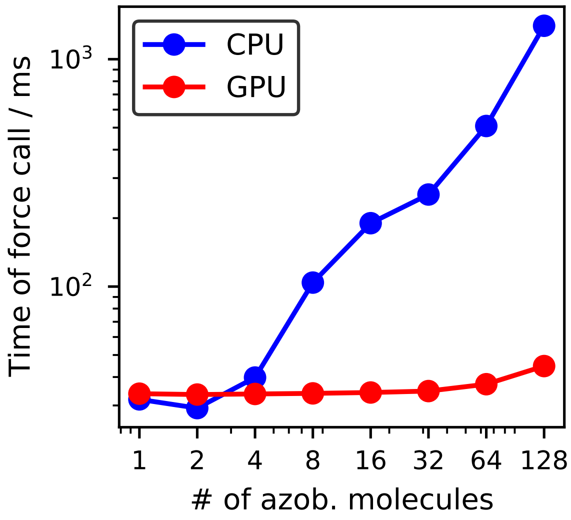

Figure S1: **GPU speed up for ANI** The timing of force calls per molecule remains constant using a GPU as long as the system fits into memory. This scaling is in sharp contrast compared to the CPU performance, which on the other hand could be sped up using parallel computing.

1.1.3 sGDML

To fit the sGDML models we used the command line tool `sgdml all` of the sGDML package.[2]

For example:

`sgdml all train1_1000.npz 925 75`

1.1.4 GAP

To fit the GAP models we used the `gap_fit` command line tool of the GAP package.[3] As a descriptor a 2B plus double SOAP was used. The 2B descriptor had a cutoff of 6 Å, the short range SOAP kernel had a 2.5 Å and the longer range SOAP kernel had 4.5 Å cutoff. For both SOAP kernels we used $n_{max} = 6$, $l_{max} = 12$ and selected 750 sparse points. The `atom_sigma` was set to 0.3 and 0.5, and the `cutoff_transition_width` to 0.5 and 1.0, for the short and long ranged SOAP respectively. The `zeta` parameter was 4, and the `delta` 0.1 for both of them.

1.1.5 Classical Force Field

The modified GAFF force field was fit using the ForceBalance program with Amber17.[4,5] Bond, angle and dihedral terms were reparametrized, whilst non-bonded and improper terms remained unchanged. Regularization was not used as overfitting is unlikely given the size of the dataset and the simplicity of the functional form. Both energies and forces were used in the fitting process and the weighting of force to energy was set to 1:1. The parameter optimization was performed using the Newton-Raphson algorithm with a search tolerance of 0.001.

1.2 Learning curves

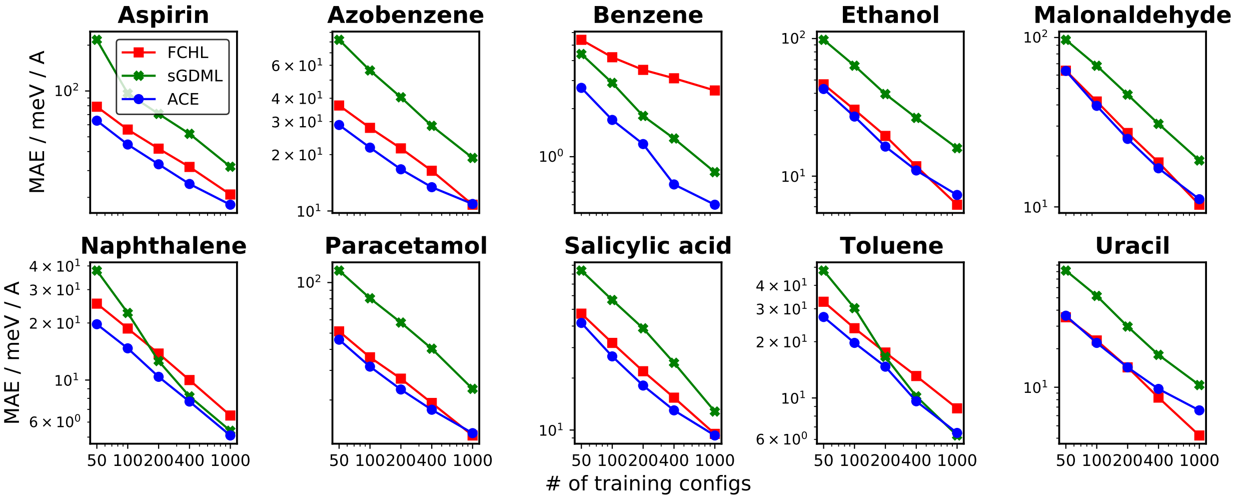

Figure S2: **Force learning curves on the MD17 dataset**

1.3 Normal mode analysis

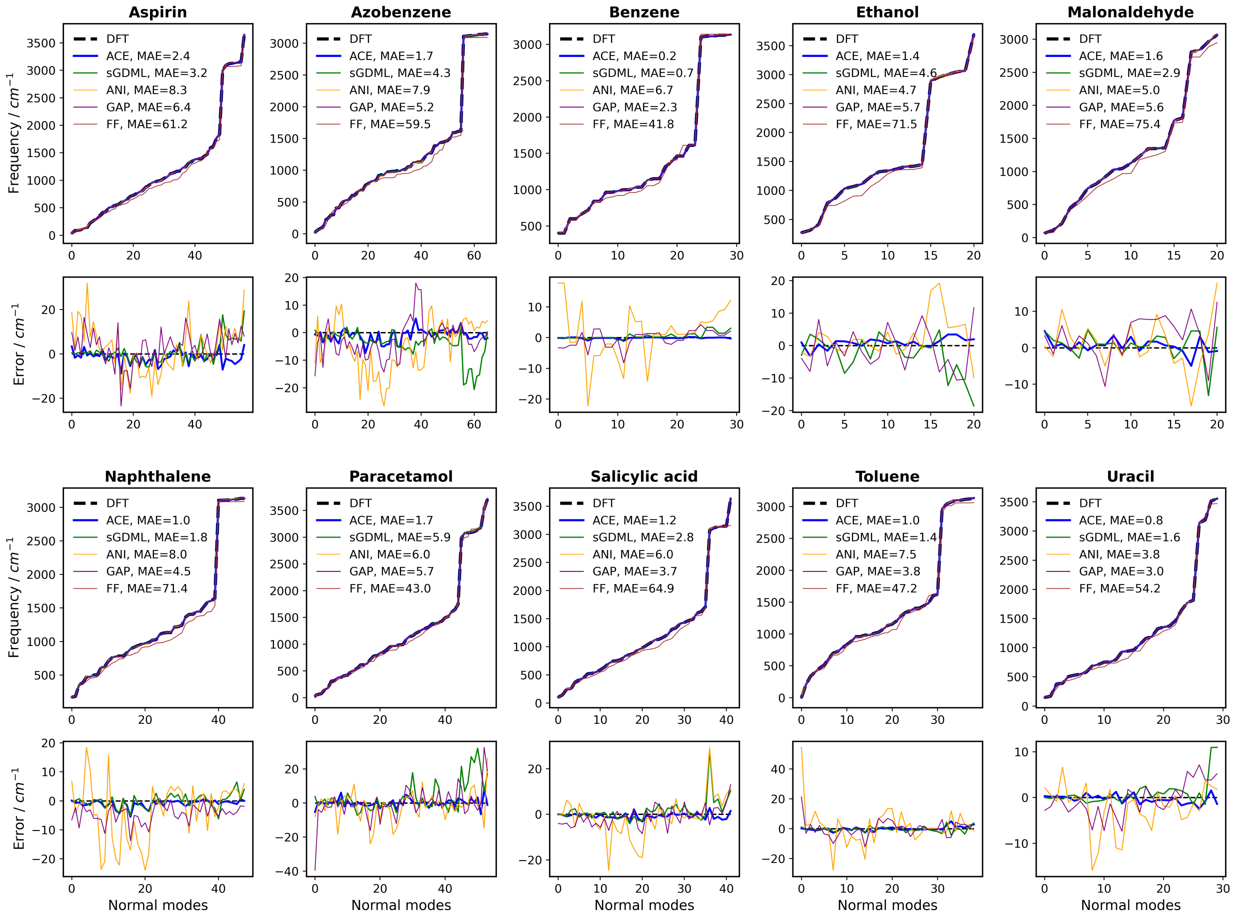

Figure S3: **Normal mode frequency test** Normal mode frequencies of the 10 MD17 molecules showing the error of the classical force field along with the ML models.

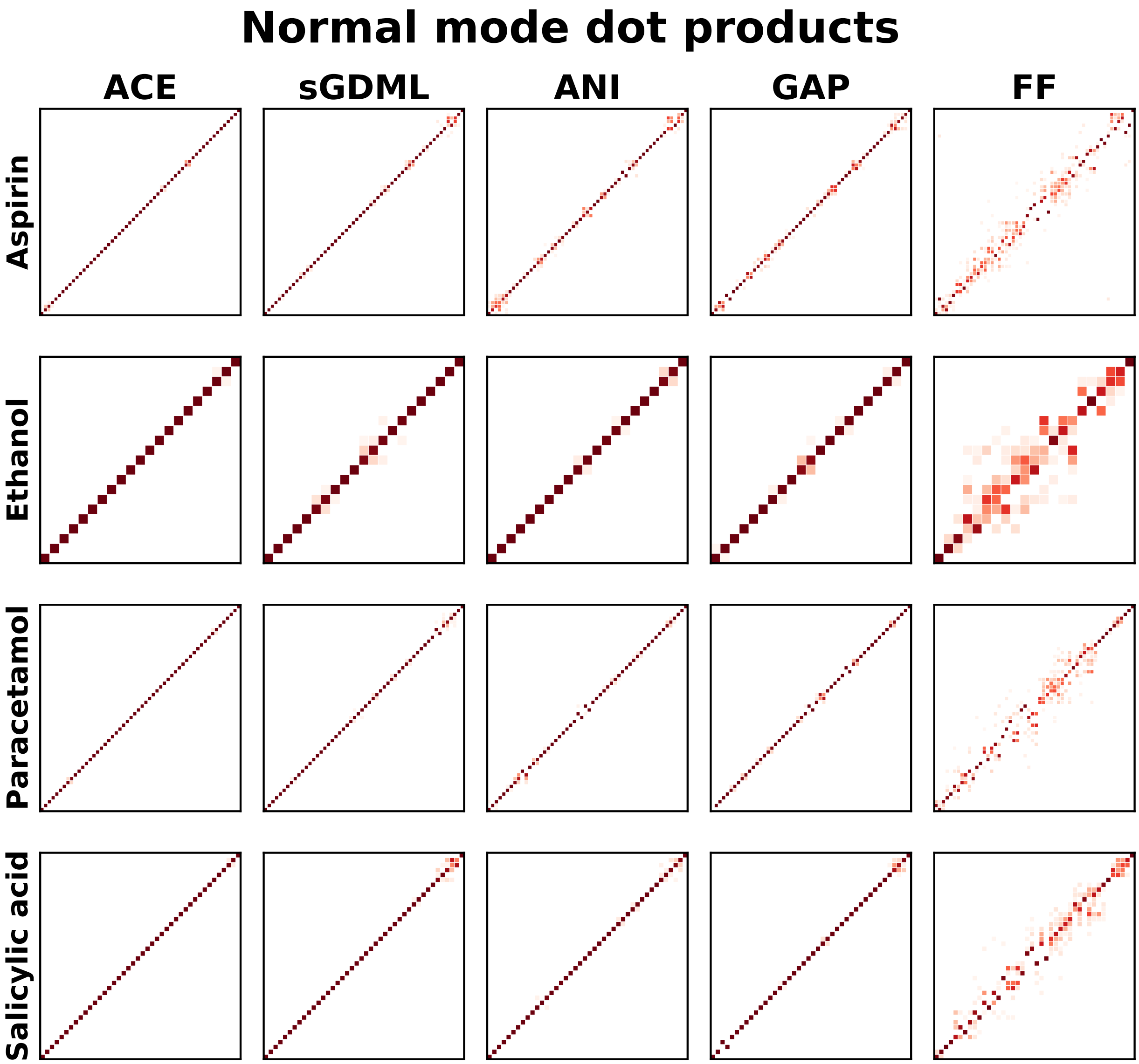

Figure S4: **Normal mode vector test** The value of the dot product of the normal modes of each of the models with the DFT (ground truths) normal mode vectors is plotted.

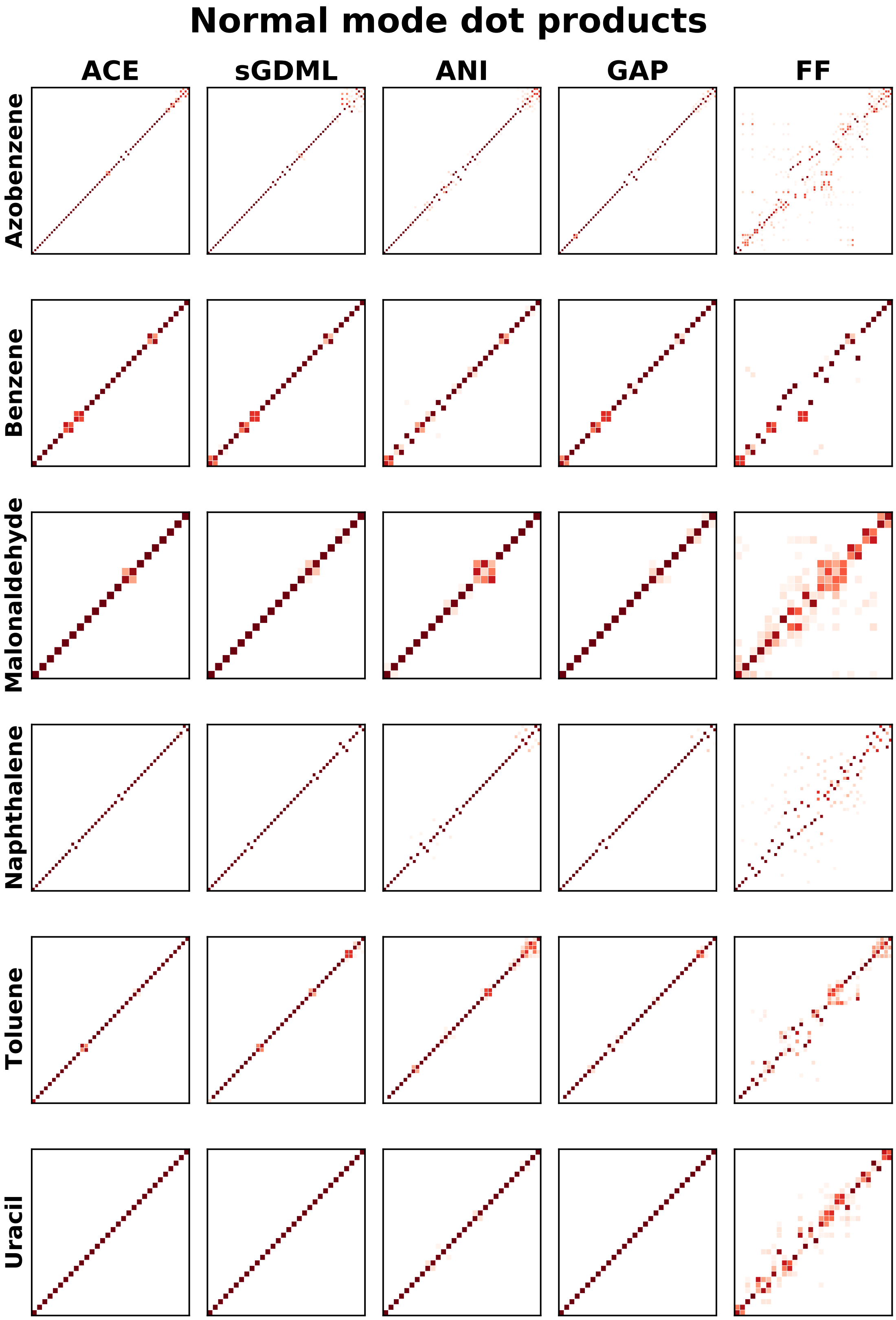

Figure S5: **Normal mode vector test** The value of the dot product of the normal modes of each of the models with the DFT (ground truths) normal mode vectors is plotted.

2 Extrapolation test

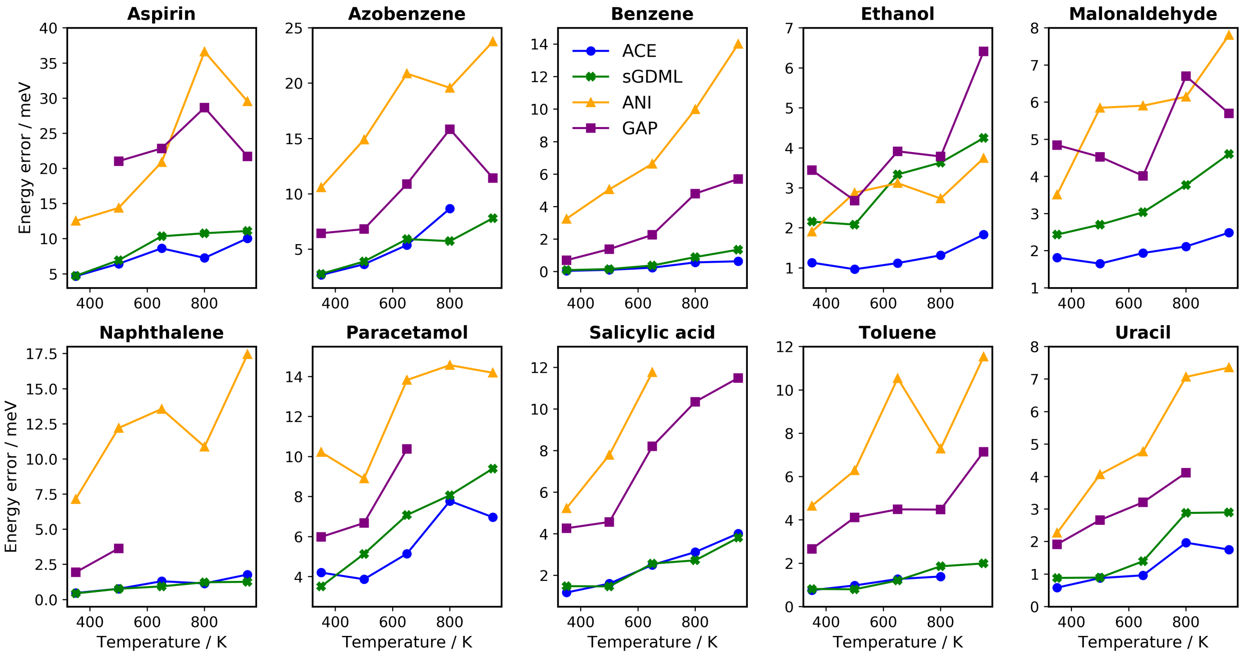

Figure S6: **Extrapolation energy error**

2.1 The effect of the isolated atom energy

Table S3: The mean absolute error of energies (meV) and forces (meV / A) of ACE models trained on energies and forces using the average energy shift, the isolated atom energy shift and trained on forces only and then shifted to minimize training energy error.

		**ACE with iso E0**	**ACE with average E0**	**ACE with forces only**
**Aspirin**	Energy	6.1	**5.9**	6.1
	Force	19.1	18.7	**17.9**
**Azobenzene**	Energy	**3.4**	3.5	3.6
	Force	12.8	14.8	**10.9**
**Benzene**	Energy	0.04	0.04	0.04
	Force	0.5	0.5	0.5
**Ethanol**	Energy	1.4	1.4	**1.2**
	Force	8.4	8.2	**7.3**
**Malonaldehyde**	Energy	1.9	1.8	**1.7**
	Force	12.0	11.5	**11.1**
**Naphthalene**	Energy	0.9	0.9	0.9
	Force	**5.1**	5.2	**5.1**
**Paracetamol**	Energy	4.0	**3.8**	4.0
	Force	14.9	13.8	**12.7**
**Salicylic acid**	Energy	2.3	2.2	**1.8**
	Force	11.2	10.5	**9.3**
**Toluene**	Energy	1.1	1.1	1.1
	Force	6.7	6.7	**6.5**
**Uracil**	Energy	1.4	1.2	**1.1**
	Force	8.7	7.8	**6.6**

3 Large flexible molecule

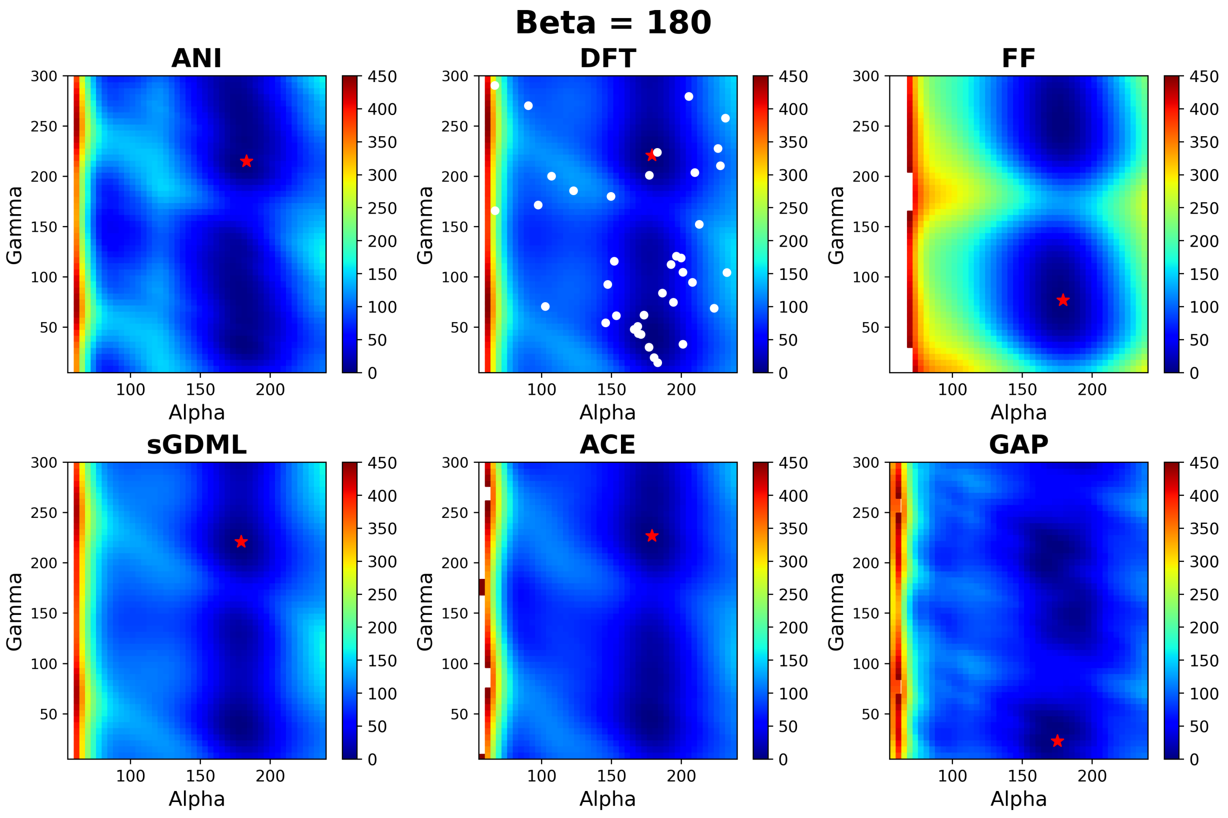

Figure S7: **Dihedral PES with** $\beta = 180^o$

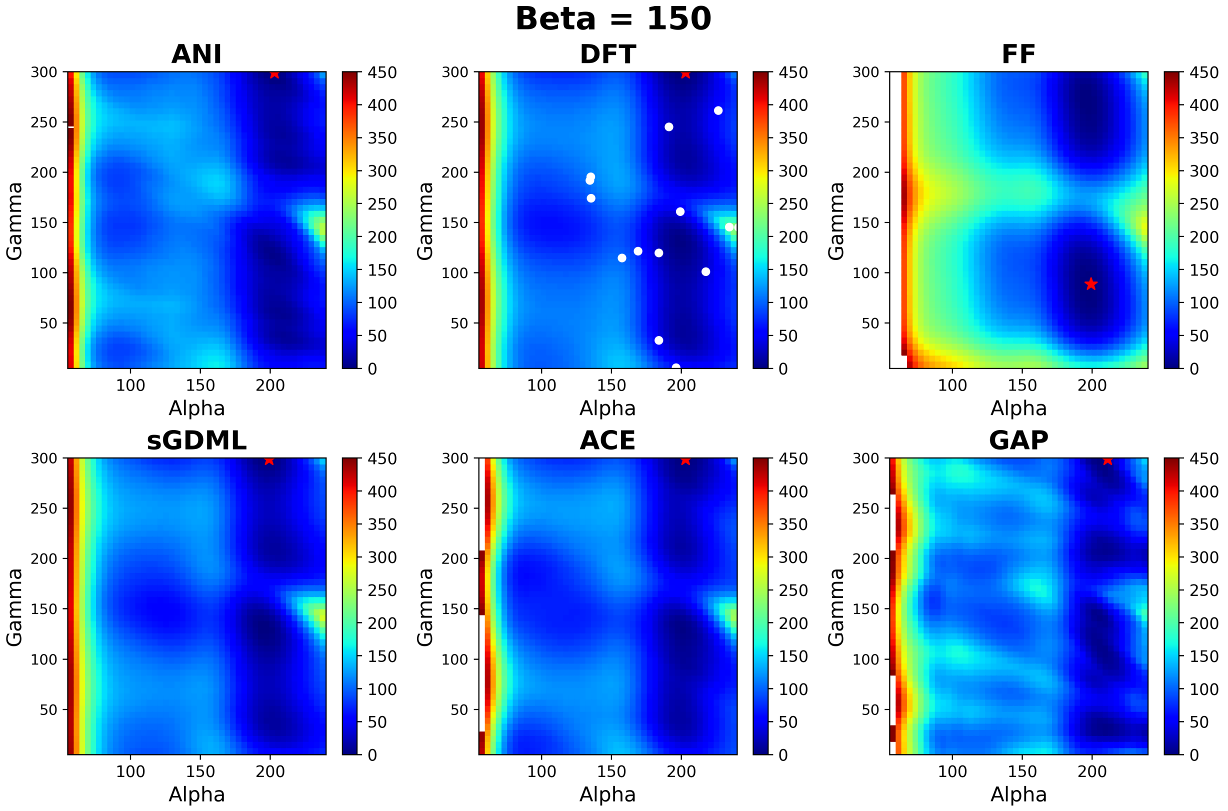

Figure S8: **Dihedral PES with $\beta = 150^o$**

References

(1) Gao, X.; Ramezanghorbani, F.; Isayev, O.; Smith, J. S.; Roitberg, A. E. TorchANI: A Free and Open Source PyTorch-Based Deep Learning Implementation of the ANI Neural Network Potentials. *Journal of Chemical Information and Modeling* **2020**, *60*, 3408–3415.

(2) Chmiela, S.; Sauceda, H. E.; Poltavsky, I.; Müller, K. R.; Tkatchenko, A. sGDML: Constructing accurate and data efficient molecular force fields using machine learning. *Computer Physics Communications* **2019**, *240*, 38–45.

(3) Bartõk, A. P.; Csányi, G. Gaussian approximation potentials: A brief tutorial introduction. *International Journal of Quantum Chemistry* **2015**, *115*, 1051–1057.

(4) Wang, L. P.; Chen, J.; Van Voorhis, T. Systematic parametrization of polarizable force fields from quantum chemistry data. *Journal of Chemical Theory and Computation* **2013**, *9*, 452–460.

(5) Case, D.; Cerutti, D.; Cheatham, T.; III, T. D.; Duke, R.; Giese, T.; Gohlke, H.; Goetz, A.; Greene, D.; Homeyer, N.; Izadi, S.; Kovalenko, A.; Lee, T.; LeGrand, S.; Li, P.; Lin, C.; Liu, J.; Luchko, T.; Luo, R.; Mermelstein, D.; Merz, K.; Monard, G.; Nguyen, H.; Omelyan, I.; Onufriev, A.; Pan, F.; Qi, R.; Roe, D.; Roitberg, A.; Sagui, C.; Simmerling, C.; Botello-Smith, W.; Swails, J.; Walker, R.; Wang, J.; Wolf, R.; Wu, X.; Xiao, L.; D.M. York,; Kollman, P. AMBER 2017. 2017.
